# A Periodic 4‐h Extension of the Dark Period Did Not Cause Long‐Term Changes in the Circadian Regulation of Photosynthesis and Sugar Levels in Lettuces

**DOI:** 10.1002/pld3.70062

**Published:** 2025-04-21

**Authors:** Cédric Dresch, Véronique Vidal, Séverine Suchail, Huguette Sallanon, Florence Charles, Vincent Truffault

**Affiliations:** ^1^ Avignon Université, UMR95 Qualisud, 84916 Avignon, France. Qualisud, Univ Montpellier, Avignon Université, CIRAD, Institut Agro, Université de La Réunion Montpellier France; ^2^ Futura Gaïa Technologies, Mas de Polvelière, Chemin du pont des îles Rodilhan France; ^3^ Biomarqueurs Environnement Santé, Institut Méditerranéen de Biodiversité et d'Ecologie marine et continentale (IMBE), UMR 7263, Université d'Avignon et des Pays du Vaucluse Pole Agrosciences 301 rue Baruch de Spinoza Avignon France; ^4^ UMR Sécurité et Qualité des Produits d’Origine Végétale (SQPOV) Avignon France

**Keywords:** carbohydrates, circadian rhythm, controlled environment agriculture, energy savings, indoor farming, photoperiod, photosynthesis, stomatal conductance

## Abstract

The photoperiod in controlled environment agriculture can be adjusted to minimize electricity consumption, even if it differs from the plant's circadian rhythm. Daily modifications of the photoperiod disrupt the plant's circadian resonance state, resulting in altered growth and yield. However, the effects of periodic, rather than daily, photoperiod adjustments remain less understood. This study aims to investigate the effects of a 4‐h extension of the dark period every 3 days on the circadian regulation of photosynthetic activity and sugar content, as well as on lettuce yield. Control lettuces were grown under a 16/8 photoperiod, while EPD lettuces (“Exceptionally long Period of Darkness”) were grown under a repeated 16/12–16/8–16/8 photoperiod pattern from the beginning to the end of cultivation. The experiment was repeated twice, and the 4‐h extension induced a loss of photosynthetic activity of 7% and 11% during the following lighting period in the first and second experiments, respectively. The yields were not affected. The stomatal conductance followed the circadian rhythm of lettuce rather than directly responding to photoperiod modifications. Furthermore, no long‐term changes in starch and sucrose content were observed. Taken together, these results show that extending the dark period by 4 h every 3 days did not cause long‐term disruption of the circadian regulation of photosynthesis and sugar levels in lettuce. These results provide new insights for optimizing light management in controlled environment agriculture, suggesting that the management of dark periods is crucial for maintaining yields and reducing energy consumption.

## 1 Introduction

Light management in controlled environment agriculture is independent of natural sunlight. The standard photoperiod applied is 16/8, consisting of 16 h of lighting and 8 h of darkness (Pennisi et al. 2020). However, the photoperiod can be adjusted to reduce electricity consumption, a major challenge in controlled environment agriculture. Photoperiod has a direct impact on growth. Indeed, when the light period is too short, growth is limited by insufficient light quantity (Kelly et al. 2020). However, increasing the photoperiod beyond 20/4 to 22/2 or 24/0 (light/dark) also reduces the fresh weight of lettuces (Silva et al. 2022). The plant's circadian rhythm plays a key role in this growth limitation, particularly through the regulation of photosynthetic activity. In fact, the net assimilation rate of CO_2_ is 67% higher in the control than in *Arabidopsis thaliana* with a defective circadian clock (*CCA1* gene overexpressed) (Dodd et al. 2005). Furthermore, net photosynthesis primarily depends on stomatal conductance, which regulates CO_2_ levels in the leaf (Hennessey and Field 1991) and is under circadian control. The stomata of *Vicia* sp., *Arachis*, and *Avena* sp. open and close following a 22‐ to 26‐h cycle under constant lighting conditions (Pallas et al. 1974; Brogårdh and Johnsson 1975; Gorton et al. 1989). Other photosynthetic parameters are also under circadian control, as genes encoding the reaction center of photosystem II cycle (Harmer et al. 2000) and the efficiency of photosystem II exhibit daily variations, which have already been measured in marine algae ( *Gonyaulax polyedra* ), *Crassulacean acid metabolism* (CAM) plants ( *Kalanchoe daigremontiana* ), *Brassicaceae* ( *Arabidopsis thaliana* ), and lettuces (*Lactuca sativa)* (Samuelsson et al. 1983; Hennessey and Field 1991; Rascher et al. 2001; Gould et al. 2009).

Photosynthesis end‐products are regulated by the circadian clock, particularly starch and sucrose metabolism (Graf et al. 2010; Scialdone et al. 2013; Sulpice et al. 2014; Fernandez et al. 2017; Mengin et al. 2017; Flis et al. 2019). Starch is essential for normal plant growth (Stitt and Zeeman 2012; Fernandez et al. 2017). Its accumulation follows a linear pattern during the day, and its nighttime degradation rate is regulated by the circadian clock, which anticipates the expected length of the night (Gibon et al. 2004; Graf et al. 2010; Martins et al. 2013; Scialdone et al. 2013; Sulpice et al. 2014). Starch is degraded into sucrose, which is linked to the circadian rhythm through *PSEUDO‐RESPONSE REGULATOR 7* (*PRR7*), involved in morning perception, and *GIGANTEA* (*GI*), a major regulator of the circadian rhythm (Dalchau et al. 2011; Haydon et al. 2013). Sucrose is exported and subsequently degraded into glucose and fructose, which contribute to the growth and maintenance of the plant (Webb and Satake 2015).

When a plant's circadian rhythm and the photoperiod coincide, the plant is in a circadian resonance state (Dodd et al. 2005), optimal for growth, as the endogenous regulation of photosynthetic activity and sugar levels matches the external conditions. Thus, continuous photoperiod modifications are deleterious when too drastic, as they cause constant disruption of the plant's circadian resonance state (Ishii et al. 1995; Kang et al. 2013; Dodd et al. 2014; Zhou et al. 2020). However, less is known about the impact of periodic adjustments of the photoperiod. Therefore, the objective of this study is to investigate how a 4‐h extension of the dark period every 3 days affects the photosynthetic activity, sugar content, and yield of lettuce. The goal is to determine whether such adjustments induce a long‐term disruption of the circadian regulation of photosynthesis and sugar levels in lettuce, providing new insights for optimizing light management in controlled environment agriculture.

## 2 Material and Methods

### 2.1 Plant Material and Growth

**FIGURE 1:**
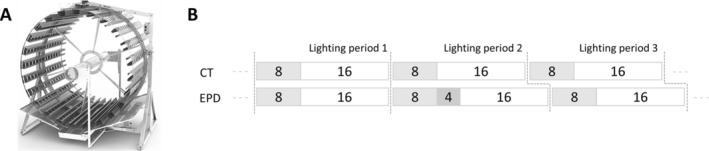
Illustration of the GiGrow cultivation system used (A) and the photoperiod applied for the control lettuces, (16/8, CT lettuces), and the “Exceptionally long Period of Darkness” modality, (16/12–16/8–16/8, EPD lettuces) (B). The lighting periods were numbered in the same way in the first experiment (Exp 1) and the second experiment (Exp 2). The 4‐h extension of the dark period in EPD lettuce, repeated every 3 days, causes a phase shift compared with the control.

Butterhead lettuce ( *Lactuca sativa* L. var. fairly, “Enza Zaden,” the Netherlands) seeds were sown in 144 (16 × 9) holes germination trays filled with potting soil (TBSP, “Florentaise,” France). The photoperiod was 16/8 (16 h of light, 8 h of darkness) with respective temperatures of 24/19°C and relative humidity of 65%. Water was provided until saturation by sub‐irrigation on a 3‐day basis with a germination solution composed of 3.2, 0.6, 0.9, 1.3, 0.8, and 0.1 mmol L^−1^ of nitrogen, phosphorus, potassium, calcium, magnesium, and sulfur, respectively. The electroconductivity (EC) of the irrigation solution was equal to 0.5 mS cm^−1^ with a pH of 5.6. Light intensity was set at 130 μmol m^2^ s^−1^ with red:blue 3:1 and ≈17% of white light, provided by LED lamps (T10 LED Grow Tube Light, HW‐GL‐T10‐1200‐36W‐3Y, China). After 14 days, plantlets were transferred to a Gigrow rotative cultivation system from Futura Gaïa Technologies (Figure 1A, France), used for industrial purposes thanks to its technical advantages, notably in terms of light homogeneity. Plants were rotating according to a horizontal rotation axis at a speed of 50 min per revolution, which provides a centrifugal force of 1.33 × 10^−5^ Newton, considered neglectable. Two experiments were conducted in July 2022 and July 2023. In the first one, lettuces were transplanted in five holes stainless steel trays (72 cm × 15 cm × 4.5 cm; length, width, height) with a density of 30 plants m^−2^. In the second experiment, lettuces were transplanted in nine holes stainless steel trays (118 cm × 15 cm × 4.7 cm; l × w × h) with a density of 39 plants m^−2^. Trays were filled with potting soil (VER4, “Florentaise,” France). The light source of the rotative cultivation system is placed at the center and is at the same distance from all plants.

Two photoperiod modalities were studied (Figure 1B):

- Control (CT) under a constant 16/8 photoperiod.
- Exceptionally long Period of Darkness (EPD) modality under a 16/12–16/8–16/8 photoperiod pattern, from the beginning to the end of cultivation.

Overall light intensity for both modalities was equal to 350 ± 28 μmol of photons m^−2^ s^−1^ provided by ceramic metal‐halide lamps (630 W double‐ended, “Lumatek Ltd,” Malta). A light specter with a 4200 K color temperature was provided, with a red:green:blue ratio of 1:0.8:0.4. Temperature and vapor pressure deficit (VPD) were set at 24°C and 1 KPa during the day and 19°C and 0.54 KPa during the night. Ambient CO_2_ concentration was not regulated. Cultivation time was set for 31 days. Irrigation solutions were made with Nutrimix of Futura Gaïa Technologies. Water supply was managed to saturate the soil in order to avoid water deficiency effects (2.5 L plant^−1^ for 31 days). The fertilization solution was composed of 29.3, 1.8, 12.5, 10, and 7.5 mmol L^−1^ of nitrogen, phosphorus, potassium, calcium, and magnesium, respectively, with an EC of 4 mS cm^−1^ and a pH of 6.5.

### 2.2 Photosynthetic Measurement Parameters

Net photosynthesis (μmol CO_2_ m^−2^ s^−1^), stomatal conductance (mmol H_2_O m^−2^ s^−1^) and the quantum yield of PSII electron transport (*ΦPSII*) were measured using a portable photosynthetic analyzer (Head Version 1.4.7, LI‐COR 6800, Li‐Cor, Inc. Lincoln, NE, USA) equipped with a LI‐COR chamber type 6800‐01. Parameters were set at 400 μmol of photons m^−2^ s^−1^ of light (red and blue light, R:B ratio of 9:1) with 420 ppm CO_2_. Temperature and VPD were not controlled and therefore remained the same as those in the cultivation chamber (24/18°C and 1/0.54 kPa for the light/dark periods). Plants were removed from the rotative cultivation system for the entire light period, maintained under similar lighting conditions, and rehydrated every hour to avoid water deficit. In the first experiment, measurements were taken during the lighting period following the 4‐h extension of the dark period for EPD lettuces, and again the following day (Lighting Periods 2 and 3 in Figure 1B). In the second experiment, measurements were taken at the same periods, additionally during the lighting period before the 4‐h extension of the dark period for EPD lettuces (Lighting Periods 1, 2, and 3 in Figure 1B). Measurements were carried out on 10 plants, with two leaves per plant, so that each leaf was measured every hour. Measurements were recorded manually every 3–5 min, with value stability serving as the indicator for recording the data. Daily photosynthesis was calculated by multiplying each net assimilation (in CO_2_ m^−2^ s^−1^) by the time interval between two measurements, resulting in a final value in μmol CO_2_ m^−2^ d^−1^.

### 2.3 Sugars Analysis

Glucose, fructose, sucrose, and starch contents were assessed at the end of lighting and dark periods from 18 to 21 days after transplantation. Results are expressed as hours after the initial sampling.

#### 2.3.1 Glucose, Fructose, and Sucrose Contents

In the first experiment, glucose, fructose, and sucrose content were assayed by high performance anion‐exchange chromatography (HPAEC). After removing the core, lettuce leaves were frozen in liquid nitrogen, grounded using IKA A11 basic Analytical mill (IKA, Germany), and then freeze‐dried using a Cryotec Cosmos freeze‐dryer (Cryotec, France). Between 15 and 20 mg of dried lettuces were sampled in ultra‐pure water to obtain a concentration of 10 mg of DW mL^−1^. Samples were homogenized and centrifuged for 4 min at 15,000*g*, 4°C (Merck 3‐16KL, KGaA, Germany). Supernatant was collected and filtered through a PTFE membrane filter (0.2 μm, IC Millex‐LG, Merck KGaA, Germany). Glucose, fructose, and sucrose contents were assayed using HPAEC (Dionex ICS‐3000, USA) by injecting 5 μL of each sample. Separation was carried out using an IC Dionex CarboPac PA1 analytical anion‐exchange column (10 μm, 4 × 250 mm, Dionex, USA). The mobile phase was a solution of H_2_O (eluent A) and 250 mM NaOH with 4 mM sodium acetate (eluent B) with an A/B ratio of 35:65 (v/v). An isocratic elution mode at a constant flow rate of 0.7 mL min^−1^ was performed. Sugars were monitored by pulsed amperometric detection (PAD), with a detection peak at 254 nm. Curves of glucose, fructose, and sucrose contents were obtained by injecting calibration standards with concentrations of 2.5, 5, 7.5, 10, 12.5, 15, and 20 nmol. CHROMELEON software v.6.7. was used to analyze the concentration of sugars. The limit of quantification was calculated according to Rusch et al. (Rusch et al. 2013). In the second experiment, glucose, fructose, and sucrose content were assayed using a sucrose/D‐fructose and D‐glucose assay kit (K‐SUFRG, Megaenzyme, Ireland). Five milligrams of dried lettuce were solubilized in 2 mL ultra‐pure water and centrifuged for 5 min at 12,000 g at 4°C before assay. For sucrose analysis, 20 μL of β‐fructosidase was added to 10 μL of the sample solution. After incubating for 5 min at 23°C, 190 μL of ultra‐pure water, 10 μL of buffer solution (Solution 1, provided), and 10 μL of NADP+/ATP solution (Solution 2, provided) were added. Absorbance was measured at 340 nm. For glucose and fructose analysis, 210 μL of ultra‐pure water, 10 μL of buffer solution (Solution 1, provided), and 10 μL of NADP+/ATP solution (Solution 2, provided) were added to 10 μL of the sample. Absorbance was measured at 340 nm. Respective blanks for the sucrose and glucose/fructose analyses were prepared by replacing the 10 μL of sample solution with ultra‐pure water. Then, 2 μL of hexokinase/D‐glucose‐6‐phosphate dehydrogenase solution (Solution 3, provided) were added to all samples. After 5 min, absorbance was measured at 340 nm. For fructose analysis, 2 μL of phosphoglucose isomerase solution (Solution 4, provided) were added, and absorbance was measured at 340 nm after 10 min. The sucrose, glucose, and fructose contents were calculated according to the kit's instructions (K‐SUFRG, Megaenzyme, Ireland).

#### 2.3.2 Starch Extraction and Starch Content Assays

Starch extraction was performed on 10 mg of dried lettuce. Samples were washed off any free glucose twice with 1 mL of 90% ethanol. They were then centrifuged for 2 min at 10,000 g, 4°C, and 1 mL of ultra‐pure water was added. Samples were autoclaved for 60 min at 121°C (ALV1131, “Getinge,” France) and weighed to determine the water loss during the autoclaving. Five hundred microliters of ultra‐pure water were added, and samples were homogenized. Starch content was determined using a starch fluorometric assay kit (MAK368, Sigma‐Aldrich, USA). Starch was hydrolyzed and revealed, and starch content was determined by fluorimetry (Synergy HT, BioTek Instruments, USA).

### 2.4 Agronomical Parameters

Fresh and dry weights were measured at eight different time points throughout the cultivation period and at harvest (31 days after transplantation). Whole plants were harvested, and fresh weight was measured using a precision balance XT 620M (PRECISA, France). Two leaves were dried in an oven at 80°C for at least 3 days to calculate the dry weight.

### 2.5 Statistical Analysis

Statistical analyses were carried out using the pairwise Wilcoxon–Mann–Whitney test (*α* = 0.05) on RStudio Version 4.2.2 (R Core Team 2022) using dplyr, ggpattern, ggplot2, ggpubr, grid, gridExtra, multcompView, rcompanion, sf, and tidyr packages. Details of all *p*‐values are presented in Supporting Information.

## 3 Results

### 3.1 Agronomic Traits Are Not Altered by Repeated 4‐h Extensions of the Dark Period Throughout the Growth Period

Fresh and dry weight data are compiled in Table 1. A similar amount of both fresh and dry weight per tray was produced, regardless of the modality or experiment. In the first experiment (Exp 1), differences in fresh weight between the two modalities were only observed at 5 and 8 days after transplantation. In the second experiment (Exp 2), differences were measured at 11, 14, and 19 days after transplantation. No reproducible differences in fresh and dry weights were observed between the two modalities.

**TABLE 1:** Mean ± standard error of fresh and dry weight per tray for CT (photoperiod: 16/8) and EPD (photoperiod pattern: 16/12–16/8–16/8) lettuce for both experiments at different days after transplantation (DAT). Each tray had five plants in the first experiment (Exp 1) and nine plants in the second experiment (Exp 2).

	Fresh weight (g per tray unit)				Dry weight (g per tray unit)		
	DAT	CT	EPD	*p*‐value	CT	EPD	*p*‐value
Exp 1 (five plants)	0	2 ± 0.3	2 ± 0.4	1.0000	0.1 ± 0.0	0.1 ± 0.0	0.3095
	5	4 ± 1	6 ± 0.3	0.0079*	0.4 ± 0.1	0.4 ± 0.0	0.1508
	8	14 ± 1	17 ± 2	0.0317*	1.1 ± 0.1	1.2 ± 0.1	0.0952
	11	48 ± 7	50 ± 6	0.5476	2.5 ± 0.3	2.9 ± 0.4	0.1508
	14	135 ± 27	137 ± 17	0.8413	6.5 ± 1.2	6.6 ± 1.1	1.0000
	19	393 ± 79	339 ± 69	0.2222	16.8 ± 2.8	15.3 ± 2.2	0.4206
	24	774 ± 153	763 ± 131	1.0000	29.8 ± 6.2	32.6 ± 5.7	0.4206
	28	1233 ± 42	1093 ± 79	0.1508	43.2 ± 3.3	36.6 ± 5.1	0.1508
	31	1387 ± 204	1418 ± 140	1.0000	44.7 ± 6.8	48.8 ± 7.0	0.3095
Exp 2 (nine plants)	0	5 ± 1	5 ± 1	1.0000	0.4 ± 0.1	0.4 ± 0.1	1.0000
	5	6 ± 1	6 ± 1	0.2032	0.6 ± 0.1	0.6 ± 0.1	0.3406
	8	15 ± 3	18 ± 4	0.1058	1.2 ± 0.2	1.3 ± 0.3	0.4776
	11	49 ± 14	37 ± 10	0.0350*	3.7 ± 0.7	2.8 ± 0.5	0.0011*
	14	157 ± 26	131 ± 21	0.0093*	9.0 ± 1.4	8.0 ± 1.2	0.0387*
	19	364 ± 51	274 ± 44	0.0001*	20.1 ± 3.7	16.5 ± 2.7	0.0121*
	24	799 ± 134	700 ± 100	0.0887	32.5 ± 6.7	31.6 ± 5.4	0.8874
	28	1333 ± 193	1152 ± 221	0.1782	64.8 ± 20.1	61.0 ± 19.7	0.7728
	31	1374 ± 120	1388 ± 149	0.8874	62.4 ± 8.0	60.3 ± 10.1	0.8874

*Note: p*‐values were calculated using the Wilcoxon–Mann–Whitney test, and significant differences are marked with asterisks (*α* = 0.05).

### 3.2 The Daily Photosynthesis Trend of EPD Lettuces Is Similar to the Control When Expressed as a Function of the Lighting Hours Anticipated by the Plants' Circadian Rhythm

**FIGURE 2:**
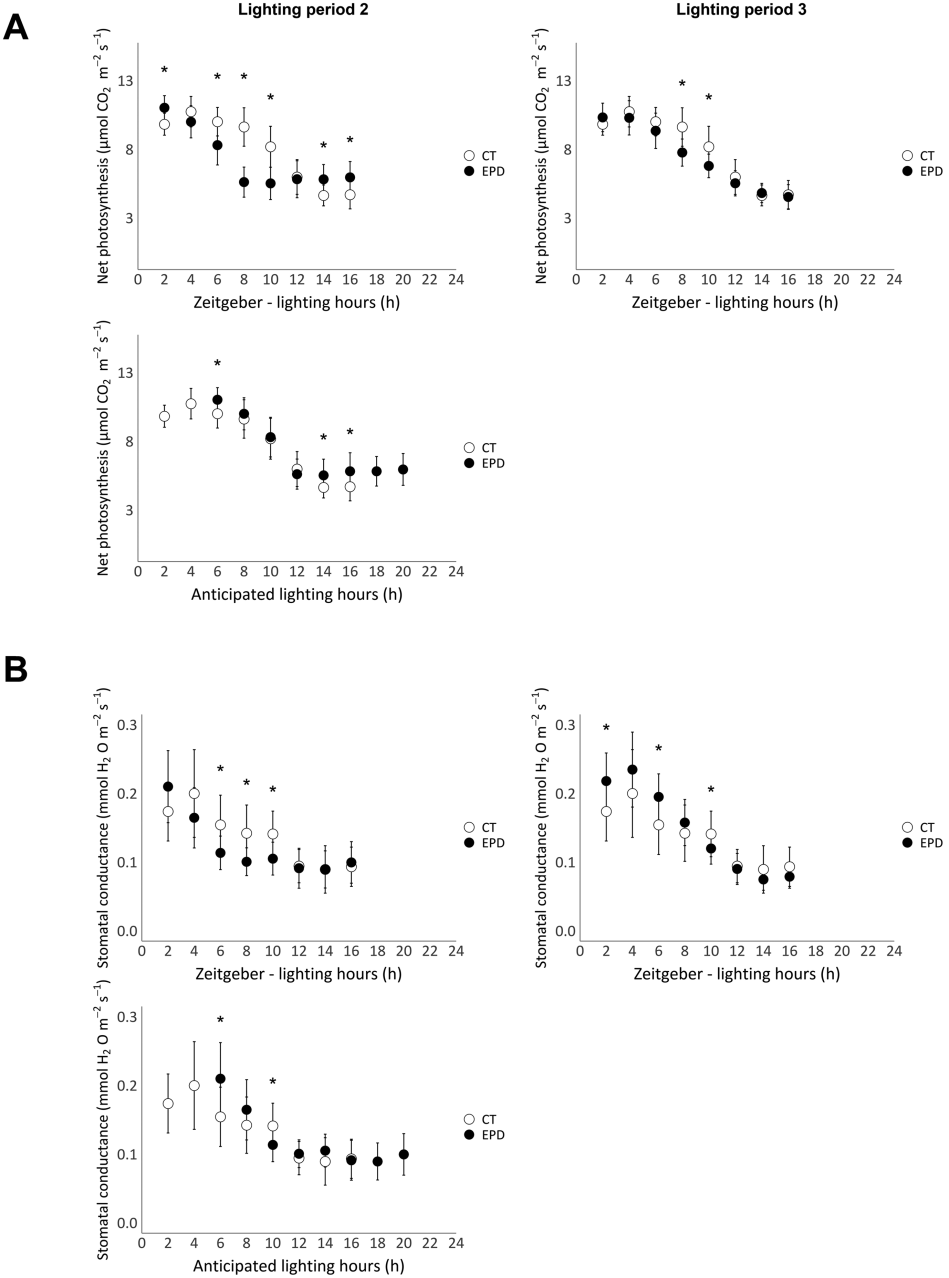
Net photosynthesis (A) and stomatal conductance (B) values of control (photoperiod: 16/8, CT lettuces) and EPD lettuces (photoperiod pattern: 16/12–16/8–16/8) of the first experiment, measured using the LI‐6800. Measurements were taken after the 4‐h extension of the dark period for EPD lettuces (Lighting Period 2), as well as the following day (Lighting Period 3). Data are expressed as a function of a zeitgeber, the hours of lighting (h), or the anticipated lighting hours by the plant, consistent with their circadian rhythm. Each data point represents the mean ± standard error of all measurements taken during the specified periods (*n* ≥ 10, on 10 plants, two leaves per plant). Statistically significant differences are indicated by asterisks (pairwise Wilcoxon–Mann–Whitney test, *α* = 0.01).

**FIGURE 3:**
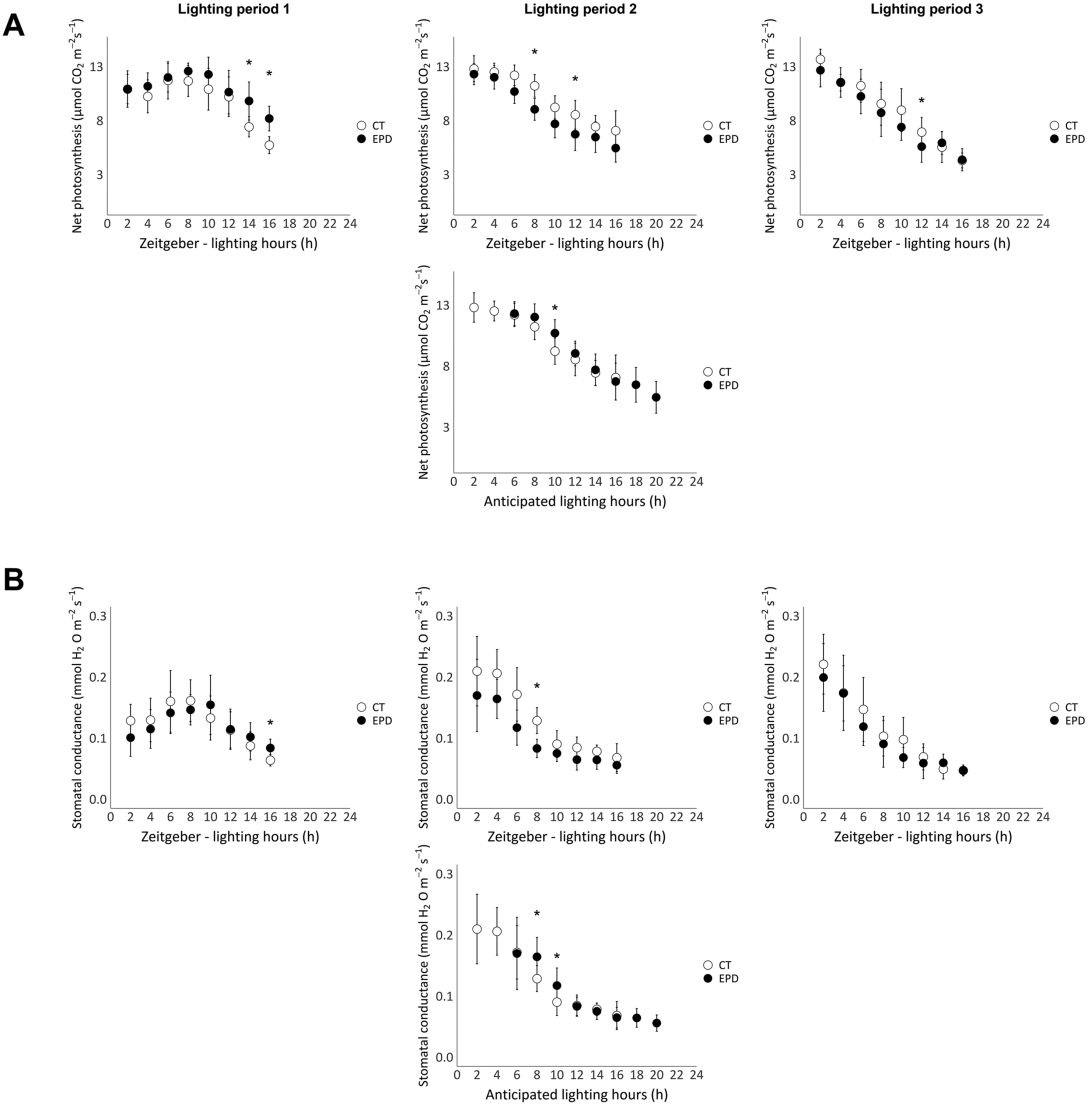
Net photosynthesis (A) and stomatal conductance (B) values of control (photoperiod: 16/8, CT lettuces) and EPD lettuces (photoperiod pattern: 16/12–16/8–16/8) of the second experiment, measured using the LI‐6800. Measurements were taken the day before (Lighting Period 1), the day after (Lighting Period 2), and the day following (Lighting Period 3) the 4‐h extension of the dark period of EPD lettuces. Data are expressed as a function of a zeitgeber, the hours of lighting (h), or the anticipated lighting hours by the plant, consistent with their circadian rhythm. Each data point represents the mean ± standard error of all measurements from two independent repetitions taken during the specified periods (*n* ≥ 10, on 2 × 10 plants, two leaves per plant). Statistically significant differences are indicated by asterisks (pairwise Wilcoxon–Mann–Whitney test, *α* = 0.01).

Figures 2 and 3 represent the net photosynthesis (A) and the stomatal conductance (B) of lettuces from the first and second experiments, respectively. In both figures, the data are presented as a function of zeitgeber time (lighting hours) or anticipated lighting hours, based on the plants' endogenous circadian rhythm.

In both experiments (Figures 2 and 3), net photosynthesis values of CT lettuces are maximal during the first half of the lighting period (from 0 to 8 h) and lowest towards the end, between 12 and 16 h. Also, during the second lighting period, EPD values are maximal only during the 4 h of lighting and then decrease in both experiments. Net photosynthesis daily trend of both modalities in the first lighting period in the second experiment and in the first and third lighting periods in both experiments are similar. For the second lighting period, after the 4‐h extension of the dark period, when net photosynthesis is expressed as a function of lighting hours, CT and EPD lettuces show different daily trends, especially in the first experiment. However, when net photosynthesis values of EPD lettuces are expressed as a function of the lighting hours anticipated by the plants' circadian rhythm, the daily trends of net photosynthesis of both modalities are more similar in both experiments.

Stomatal conductance values follow the same patterns as net photosynthesis in both experiments but show more variability.

### 3.3 Starch and Sucrose Contents Varied According to the Photoperiod and Responded to the 4‐h Extension of the Dark Period

**FIGURE 4:**
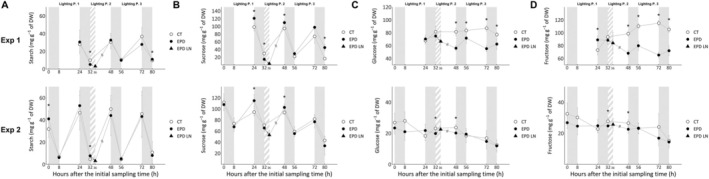
Carbohydrates contents of control (photoperiod: 16/8, CT lettuces) and EPD lettuces (photoperiod pattern: 16/12–16/8–16/8) over 3 days. Data from the first experiment are shown in row “Exp 1,” and data from the second experiment are shown in row “Exp 2.” Data are expressed as mg g^−1^ of dry weight (DW). Time is expressed as hours after the initial sampling time (h). White backgrounds represent lighting periods, gray backgrounds represent dark periods, and striped backgrounds represent dark periods for EPD lettuces only (control lettuces under lighting). A double slash for EPD lettuces marks the omission of 4 h of lighting for clarity in representation. (A) Starch content. (B) Sucrose content. (C) Glucose content. (D) Fructose content. Each symbol represents the mean ± standard error of five samples in the first experiment and the mean ± standard error of 2 × 3 samples from two independent repetitions in the second experiment. Statistically significant differences are indicated by asterisks (pairwise Wilcoxon–Mann–Whitney test, *α* = 0.05).

Starch, sucrose, glucose, and fructose contents of CT and EPD lettuces from both experiments are represented in Figure 4.

Starch (Figure 4A) and sucrose (Figure 4B) contents in both experiments, for both CT and EPD modalities, vary according to the photoperiod. Values are maximal at the end of the lighting period and minimal at the end of the dark period. In both experiments, the extended 4‐h dark period for EPD lettuces induces a decrease in starch and sucrose contents compared with their levels after the 8‐h dark period (dark period before the Lighting Period 2). In the first experiment, the starch and sucrose contents of EPD lettuces after a 12‐h dark period are three and nine times lower, respectively, than those of the control after an 8‐h dark period. In the second experiment, the starch and sucrose contents of EPD lettuces after a 12‐h dark period are 1.5 and 1.3 times lower, respectively, than those of the control after an 8‐h dark period.

Glucose (Figure 4C) and fructose (Figure 4D) did not vary as significantly as sucrose and starch contents. Moreover, in the first experiment, glucose and fructose contents of EPD lettuces are lower than those of the control, while in the second experiment, glucose and fructose contents of EPD lettuces are similar to those of the control.

## 4 Discussion

### 4.1 The Circadian Rhythm of Stomatal Conductance Limits Photosynthesis After Periodic 4‐h Extensions of the Dark Period, With No Impact on Final Yields

In this experiment, net photosynthesis is maximal at the beginning of the lighting period and decreases throughout the day, consistent with patterns previously described in *Symbodinium* sp., *Gonyaulax polyedra* , *Phaseolus vulgaris* (common bean), *Marchantia polymorpha*, and *Zantedeschia aethiopica* (Hastings et al. 1961; Fredeen et al. 1991; Sorek et al. 2013; Cuitun‐Coronado et al. 2022; Dias et al. 2022). The same pattern can be observed for stomatal conductance. Indeed, net photosynthesis and stomatal conductance are decreasing synchronously. We can precise that net photosynthesis is related to stomatal conductance by a logarithmic relationship, with a higher correlation coefficient of 0.89 for CT lettuces and 0.88 for EPD lettuces in the second experiment (Figure S1), consistent with previous studies (Gorton et al. 1989; Gago et al. 2016). In these experiments, extending the dark period by 4 h every 3 days induced a 4‐h shift in the daily trend of net photosynthesis and stomatal conductance values expressed as a function of the lighting hours. However, this extension did not affect the daily trend when the values were expressed as a function of the lighting hours anticipated by the plant's circadian rhythm. Our results are consistent with literature that previously described that stomatal conductance is under circadian regulation (Kreps and Kay 1997) and showed that this regulation is not disrupted by a periodic extension of the dark period. Thus, net photosynthesis is also regulated by the circadian rhythm and not directly by the photoperiod. It should be noted that other photosynthetic parameters, such as the efficiency of the photosystem II (*ΦPSII*, Figure S2), already known to be regulated by an endogenous circadian rhythm through specific gene expression (Harmer et al. 2000; Dodd 2005; Dodd et al. 2014), are also decreasing following a daily trend. However, in this experiment, stomatal conductance consistently decreased before *ΦPSII* and other photosynthetic parameters, highlighting its specific relevance in studies of photosynthesis under modified photoperiods.

In this experiment, the dark period was extended by 4 h, but all lighting periods stayed similar to the control of 16 h. We calculated the daily net photosynthesis by multiplying each net assimilation value (in μmol CO₂ m^−2^ s^−1^) by the time interval between two measurements, resulting in a final value expressed in μmol CO₂ m^−2^ d^−1^. After the 4‐h extension of the dark period, the daily photosynthesis of EPD lettuces was 7% lower than the control in the first experiment (approximately 118 and 127 CO₂ m^−2^ d^−1^, respectively) and 11% lower than the control in the second experiment (approximately 142 and 161 CO₂ m^−2^ d^−1^, respectively). Thus, even though the duration of the lighting periods was equal in both modalities, the net photosynthesis of the control was more efficient than that of the EPD lettuces. This difference can be attributed to the duration of maximal photosynthetic activity. In control lettuces, net photosynthesis was maximal during the first 8 h of the lighting period, while in EPD lettuces, it was only maximal during the first 4 h. In EPD lettuces, the duration of maximal photosynthesis was shortened because the photoperiod was shifted by 4 h and no longer matched with the timing of maximal stomatal opening. As a result, 4 h of highly efficient photosynthesis at the beginning of the predicted lighting period were replaced by 4 h of less efficient photosynthesis at the end of the lighting period, leading to a decrease in daily net assimilation (for 16 h of lighting day^−1^). These observations are consistent with previous studies showing that the net CO₂ assimilation rate decreases when the photoperiod does not match the plant's circadian rhythm (Dodd et al. 2005). In the experiments conducted, the loss of net CO_2_ assimilation did not affect fresh and dry yields. Indeed, the fresh weight and dry weight of EPD lettuces were similar to those of the control. However, previous studies showed that a photoperiod not tuned with the circadian rhythm induces a reduction of photosynthetic activity. Indeed, Dodd et al. (2005) showed that *Arabidopsis thaliana* fixed more CO_2_ with a circadian rhythmicity of 24 h (12/12) than with days of 28 h (14/14) or 20 h (10/10). The same pattern has been observed when *Arabidopsis thaliana* expressing a circadian rhythm of 20 h were placed under long photoperiods (28 h) or when *Arabidopsis thaliana* expressing a circadian rhythm of 28 h were placed under short photoperiods of 20 h (Dodd et al. 2005). Moreover, in *Arabidopsis thaliana* , Graf et al. (2010) have measured a 37% decrease in fresh weight when the length of the day reaches 28 h (14/14) instead of 24 h (12/12). In the literature, the decrease in photosynthetic activity is higher than in our experiment, consistent with the fact that we only modified the photoperiod by 4 h on a 3‐day basis. Moreover, other experiments applied daily changes in photoperiod, which may affect plants more strongly than the periodic 4‐h extension of the dark period applied in this experiment. Finally, in the literature, extended periods of darkness might have induced a transitory depletion state of carbohydrates at the end of the darkness period, which has been assumed to inhibit growth (Gibon et al. 2004; Graf et al. 2010). In the experiments conducted, we can propose that the yield of lettuces was not altered by the disruption of the circadian rhythm because the pool of carbohydrates was not exhausted at the end of the extended dark periods.

### 4.2 Periodic 4‐h Extensions of the Dark Period Do Not Lead to Long‐Term Changes in Starch and Sucrose Regulation or Content

Starch varies according to the photoperiod in both CT and EPD modalities. Starch level increased during the lighting period and decreased during the dark periods. When a dark period of 12 h was applied, the starch level in EPD lettuces continued to decline. It can be noted that the initial amount of starch in CT and EPD lettuces was the same, as no change in lighting hours occurred. After a 12‐h dark period, 88% and 94% of the starch in EPD lettuces was consumed in the first and second experiments, respectively, consistent with starch consumption observed in *Arabidopsis thaliana* (Sulpice et al. 2014), and higher than the respective control consumption (63% and 90%, respectively).

The increase in starch consumption can be linked to the mismatch between the photoperiod and the circadian rhythm of lettuce. Indeed, when the photoperiod and the circadian rhythm are tuned, the rate of starch degradation during different night lengths is adjusted to ensure that the same amount of starch remains at the end of each dark period (Gibon et al. 2004; Bläsing et al. 2005; Graf et al. 2010; Scialdone et al. 2013; Sulpice et al. 2014; Fernandez et al. 2017). In this experiment, the starch degradation rate has not been adjusted, suggesting that the circadian rhythm of the lettuces did not anticipate the 3‐day basis dark period extension applied.

Despite the lower starch content at the end of the 12‐h dark period, the starch content in EPD lettuces during and at the end of the following light period was similar to that of the control. In the second experiment, starch accumulation during this light period in CT and EPD lettuces followed a linear trend, with correlation coefficients of 0.96 and 0.99, respectively (Figure S3). Moreover, no strong differences in starch content were found the following days. Finally, we observed that sucrose content followed the same trends as starch content. This is consistent with the fact that starch is degraded during the night to provide substrates for sucrose synthesis (Martins et al. 2013). Then, this experiment shows that a 4‐h extension of the dark period every 3 days does not lead to long‐term changes in the regulation of starch and sucrose content. This also suggests that the 16‐h light period allows enough photosynthesis to restore sugar levels to control‐like levels following a 4‐h dark period extension.

Glucose and fructose levels fluctuate less than those of starch and sucrose across light and dark periods, consistent with the fact that starch and sucrose are storage carbohydrates. In the first experiment, glucose and fructose levels of EPD lettuces were lower than the control. On the second one, glucose and fructose levels of CT and EPD lettuces are similar. Furthermore, glucose and fructose levels in the first experiment were higher than those in the second, a difference that does not seem attributed to starch and sucrose content, as these levels were similar between the two experiments. The observed variation may instead be explained by differences in growth. The second experiment used a higher planting density, which led to lower growth rates for the lettuces compared with the first experiment but resulted in the same production per tray. Additionally, the higher planting density led to partial leaf overlap, which may have also influenced glucose and fructose accumulation.

Sugar levels are under circadian control (Harmer et al. 2000), but also regulate the circadian rhythm of plants, with a key role of starch and sucrose (Webb and Satake 2015). However, in this experiment, no long‐term effects of periodic 4‐h extensions of the dark period on starch and sucrose levels were observed, suggesting that such extensions do not induce long‐term changes in the circadian rhythm of lettuces through sugar levels.

## 5 Conclusion

In this work, we studied the effects of modifications of the photoperiod by increasing the darkness period on a 3‐day basis while maintaining the same lighting period (16/12–16/8–16/8, repeated). Our measurements showed that stomatal conductance in lettuces was regulated by the plant's endogenous circadian rhythm and did not directly respond to the 4‐h extension of the dark period. Because stomatal conductance is a key limiting factor for net photosynthesis, net photosynthesis was also regulated by the plant's circadian rhythm. Because stomatal conductance and net photosynthesis were highest at the start of the light period, the extended dark period reduced daily photosynthetic activity by 7% and 11% in experiments one and two, respectively. However, fresh and dry yields were unaffected. Although starch and sucrose levels initially dropped below control levels following the extended dark period, a single 16‐h light period was sufficient to restore starch and sucrose to control‐like levels, with no differences observed the following day. This supports the idea that extending the dark period of 4 h every 3 days does not cause long‐term changes in starch and sucrose content. When combining data on stomatal conductance and sugar regulation, we can conclude that a 4‐h extension of the dark period every 3 days does not cause long‐term changes in the circadian regulation of photosynthesis and sugar levels.

This experiment provides two new insights for optimizing light management in controlled environment agriculture. First, sugar levels in lettuces exposed to an extended dark period returned to control‐like levels after a single 16‐h light period, suggesting that 16 h of light are sufficient to saturate sugar stores in the studied lettuce. However, the standard photoperiod in controlled environment agriculture is typically 16/8, which may need to be reconsidered to optimize light efficiency. Second, our results indicate that it may be more efficient to turn off lighting at the end of the light period when photosynthetic activity is at its lowest. Further experiments are needed to confirm whether shortening the lighting period impacts the plant's circadian rhythm in the same way as extending the dark period.

The main perspective of this study is that periodic photoperiod modifications may not disrupt the plant's circadian resonance state, although further research is needed to determine the maximum phase shift that can be applied without causing disruption. This will provide new insights to further optimize light management in controlled environment agriculture.

## Author Contributions

V.T., H.S., and F.C. initiated and conceived the project. C.D. created the protocol. V.T. and F.C. revised the protocol. C.D. performed the experiments and collected the materials. C.D. and V.V. performed starch assays. C.D. and S.S. performed glucose, fructose, and sucrose assays. C.D. performed the data analysis. C.D. wrote the article. V.T., F.C., and H.S. revised the manuscript. All the authors read and approved the final manuscript.

## Conflicts of Interest

The authors declare no conflicts of interest.

## Peer Review

The peer review history for this article is available in the Supporting Information for this article.

## Supporting information


**Data S1.** Peer review.

**Figure S1.** The relationship between net photosynthesis and stomatal conductance in control lettuces (photoperiod: 16/8, CT lettuces) and EPD lettuces (photoperiod pattern: 16/12–16/8–16/8) during the first and second experiments.

**Figure S2.** Efficiency of the photosystem II (*ΦPSII*) values of control (photoperiod: 16/8, CT lettuces) and EPD lettuces (photoperiod pattern: 16/12–16/8–16/8) measured using the LI‐6800 and represented across hours of the day. In “Exp 1,” measurements were taken after the 12‐h dark period (Lighting Period 2) and the following day (Lighting Period 3). In “Exp 2,” measurements were taken the day before (Lighting Period 1), the day after (Lighting Period 2), and the day following the 12‐h dark period for EPD lettuces (Lighting Period 3). White backgrounds represent lighting periods, dark gray backgrounds represent dark periods, light gray backgrounds represent dark periods for control lettuces only (EPD lettuces under lighting), and striped backgrounds represent dark periods for EPD lettuces only (control lettuces under lighting). In “Exp 1,” each data point represents the mean ± standard error of all measurements taken during the specified periods (*n* ≥ 10). In “Exp 2,” each data point represents the mean ± standard error of all measurements from two independent repetitions taken during the specified periods (*n* ≥ 10). Statistically significant differences are indicated by asterisks (pairwise Wilcoxon–Mann–Whitney test, *α* = 0.01).

**Figure S3.** Starch accumulation of control (photoperiod: 16/8, CT lettuces) and EPD lettuces (photoperiod pattern: 16/12–16/8–16/8) throughout the lighting period. Data were only collected in the second experiment. The origin of the x axis (value = 0) represents the end of an 8‐h dark period for CT lettuces and the end of a 12‐h dark period for EPD lettuces. Each symbol represents the mean ± standard error of 2 × 3 samples from two independent repetitions.

**Data S1.** Supporting information.

## Data Availability

All data presented in the article are available at Zenodo, DOI: https://doi.org/10.5281/zenodo.15058505. Data will be shared upon request from the corresponding author, Cédric Dresch (cedric.dresch@futuragaia.com).

## References

[pld370062-bib-0001] Bläsing, O. E. , Y. Gibon , M. Günther , et al. 2005. “Sugars and Circadian Regulation Make Major Contributions to the Global Regulation of Diurnal Gene Expression in *Arabidopsis* .” Plant Cell 17: 3257–3281. 16299223 10.1105/tpc.105.035261 PMC1315368

[pld370062-bib-0002] Brogårdh, T. , and A. Johnsson . 1975. “Regulation of Transpiration in Avena. Responses to White Light Steps.” Physiologia Plantarum 35: 115–125.

[pld370062-bib-0003] Cuitun‐Coronado, D. , H. Rees , J. Colmer , A. Hall , L. L. de Barros Dantas , and A. N. Dodd . 2022. “Circadian and Diel Regulation of Photosynthesis in the Bryophyte *Marchantia polymorpha* .” Plant, Cell & Environment 45: 2381–2394. 10.1111/pce.14364 PMC9546472 35611455

[pld370062-bib-0004] Dalchau, N. , S. J. Baek , H. M. Briggs , et al. 2011. “The Circadian Oscillator Gene *GIGANTEA* Mediates a Long‐Term Response of the *Arabidopsis thaliana* Circadian Clock to Sucrose.” Proceedings of the National Academy of Sciences of the United States of America 108: 5104–5109. 21383174 10.1073/pnas.1015452108 PMC3064355

[pld370062-bib-0005] Dias, M. G. , T. I. da Silva , R. R. P. Cruz , L. B. Barbosa , J. C. Zanuncio , and J. A. S. Grossi . 2022. “Daily Photosynthetic Course of Calla Lily Plants.” Revista De Agricultura Neotropical 9: 6962–6962.

[pld370062-bib-0006] Dodd, A. N. 2005. “Plant Circadian Clocks Increase Photosynthesis, Growth, Survival, and Competitive Advantage.” Science 309: 630–633. 16040710 10.1126/science.1115581

[pld370062-bib-0007] Dodd, A. N. , J. Kusakina , A. Hall , P. D. Gould , and M. Hanaoka . 2014. “The Circadian Regulation of Photosynthesis.” Photosynthesis Research 119: 181–190. 23529849 10.1007/s11120-013-9811-8

[pld370062-bib-0008] Dodd, A. N. , N. Salathia , A. Hall , et al. 2005. “Plant Circadian Clocks Increase Photosynthesis, Growth, Survival, and Competitive Advantage.” Science 309: 630–633. 16040710 10.1126/science.1115581

[pld370062-bib-0009] Fernandez, O. , H. Ishihara , G. M. George , et al. 2017. “Leaf Starch Turnover Occurs in Long Days and in Falling Light at the End of the Day.” Plant Physiology 174: 2199–2212. 28663333 10.1104/pp.17.00601 PMC5543966

[pld370062-bib-0010] Flis, A. , V. Mengin , A. A. Ivakov , et al. 2019. “Multiple Circadian Clock Outputs Regulate Diel Turnover of Carbon and Nitrogen Reserves.” Plant, Cell & Environment 42: 549–573. 10.1111/pce.13440 30184255

[pld370062-bib-0011] Fredeen, A. L. , T. L. Hennessey , and C. B. Field . 1991. “Biochemical Correlates of the Circadian Rhythm in Photosynthesis in *Phaseolus vulgaris* .” Plant Physiology 97: 415–419. 16668402 10.1104/pp.97.1.415 PMC1081014

[pld370062-bib-0012] Gago, J. , D. de Menezes Daloso , C. M. Figueroa , J. Flexas , A. R. Fernie , and Z. Nikoloski . 2016. “Relationships of Leaf Net Photosynthesis, Stomatal Conductance, and Mesophyll Conductance to Primary Metabolism: A Multispecies Meta‐Analysis Approach.” Plant Physiology 171: 265–279. 26977088 10.1104/pp.15.01660 PMC4854675

[pld370062-bib-0013] Gibon, Y. , O. E. Bläsing , N. Palacios‐Rojas , et al. 2004. “Adjustment of Diurnal Starch Turnover to Short Days: Depletion of Sugar During the Night Leads to a Temporary Inhibition of Carbohydrate Utilization, Accumulation of Sugars and Post‐Translational Activation of ADP‐Glucose Pyrophosphorylase in the Following Light Period.” Plant Journal 39: 847–862. 10.1111/j.1365-313X.2004.02173.x 15341628

[pld370062-bib-0014] Gorton, H. L. , W. E. Williams , M. E. Binns , C. N. Gemmell , E. A. Leheny , and A. C. Shepherd . 1989. “Circadian Stomatal Rhythms in Epidermal Peels from *Vicia faba* .” Plant Physiology 90: 1329–1334. 16666931 10.1104/pp.90.4.1329 PMC1061891

[pld370062-bib-0015] Gould, P. D. , P. Diaz , C. Hogben , et al. 2009. “Delayed Fluorescence as a Universal Tool for the Measurement of Circadian Rhythms in Higher Plants.” Plant Journal 58: 893–901. 10.1111/j.1365-313X.2009.03819.x 19638147

[pld370062-bib-0016] Graf, A. , A. Schlereth , M. Stitt , and A. M. Smith . 2010. “Circadian Control of Carbohydrate Availability for Growth in *Arabidopsis* Plants at Night.” PNAS 107: 9458–9463. 20439704 10.1073/pnas.0914299107 PMC2889127

[pld370062-bib-0017] Harmer, S. L. , J. B. Hogenesch , M. Straume , et al. 2000. “Orchestrated Transcription of Key Pathways in *Arabidopsis* by the Circadian Clock.” Science 290: 2110–2113. 11118138 10.1126/science.290.5499.2110

[pld370062-bib-0018] Hastings, J. W. , L. Astrachan , and B. M. Sweeney . 1961. “A Persistent Daily Rhythm in Photosynthesis.” Journal of General Physiology 45: 69–76. 13712193 10.1085/jgp.45.1.69 PMC2195161

[pld370062-bib-0019] Haydon, M. J. , O. Mielczarek , F. C. Robertson , K. E. Hubbard , and A. A. R. Webb . 2013. “Photosynthetic Entrainment of the *Arabidopsis thaliana* Circadian Clock.” Nature 502: 689–692. 24153186 10.1038/nature12603 PMC3827739

[pld370062-bib-0020] Hennessey, T. L. , and C. B. Field . 1991. “Circadian Rhythms in Photosynthesis: Oscillations in Carbon Assimilation and Stomatal Conductance Under Constant Conditions.” Plant Physiology 96: 831–836. 10.1104/pp.96.3.831. 16668261 PMC1080851

[pld370062-bib-0021] Ishii, M. , T. Ito , T. Maruo , K. Suzuki , and K. Matsuo . 1995. “Plant Growth and Physiological Characters of Lettuce Plants Grown Under Artificial Light of Different Irradiating Cycles.” Environmental Control in Biology 33: 143–149.

[pld370062-bib-0022] Kang, J. H. , S. KrishnaKumar , S. L. S. Atulba , B. R. Jeong , and S. J. Hwang . 2013. “Light Intensity and Photoperiod Influence the Growth and Development of Hydroponically Grown Leaf Lettuce in a Closed‐Type Plant Factory System.” Horticulture, Environment and Biotechnology 54: 501–509.

[pld370062-bib-0023] Kelly, N. , D. Choe , Q. Meng , and E. S. Runkle . 2020. “Promotion of Lettuce Growth Under an Increasing Daily Light Integral Depends on the Combination of the Photosynthetic Photon Flux Density and Photoperiod.” Scientia Horticulturae 272: 109565.

[pld370062-bib-0024] Kreps, J. , and S. Kay . 1997. “Coordination of Plant Metabolism and Development by the Circadian Clock.” Plant Cell 9: 1235–1244. 12237384 10.1105/tpc.9.7.1235 PMC156994

[pld370062-bib-0025] Martins, M. C. M. , M. Hejazi , J. Fettke , et al. 2013. “Feedback Inhibition of Starch Degradation in Arabidopsis Leaves Mediated by Trehalose 6‐Phosphate.” Plant Physiology 163: 1142–1163. 24043444 10.1104/pp.113.226787 PMC3813640

[pld370062-bib-0026] Mengin, V. , E.‐T. Pyl , T. A. Moraes , et al. 2017. “Photosynthate Partitioning to Starch in *Arabidopsis thaliana* is Insensitive to Light Intensity but Sensitive to Photoperiod due to a Restriction on Growth in the Light in Short Photoperiods.” Plant, Cell & Environment 40: 2608–2627. 10.1111/pce.13000 28628949

[pld370062-bib-0027] Pallas, J. E. , Y. B. Samish , and C. M. Willmer . 1974. “Endogenous Rhythmic Activity of Photosynthesis, Transpiration, Dark Respiration, and Carbon Dioxide Compensation Point of Peanut Leaves.” Plant Physiology 53: 907–911. 16658814 10.1104/pp.53.6.907 PMC541472

[pld370062-bib-0028] Pennisi, G. , F. Orsini , M. Landolfo , et al. 2020. “Optimal Photoperiod for Indoor Cultivation of Leafy Vegetables and Herbs.” European Journal of Horticultural Science 85: 329–338.

[pld370062-bib-0029] R Core Team . 2022. R: A Language and Environment for Statistical Computing. R Foundation for Statistical Computing. https://www.R‐project.org/.

[pld370062-bib-0030] Rascher, U. , M.‐T. Hütt , K. Siebke , B. Osmond , F. Beck , and U. Lüttge . 2001. “Spatiotemporal Variation of Metabolism in a Plant Circadian Rhythm: The Biological Clock as an Assembly of Coupled Individual Oscillators.” Proceedings of the National Academy of Sciences of the United States of America 98: 11801–11805. 11573013 10.1073/pnas.191169598 PMC58811

[pld370062-bib-0031] Rusch, A. , S. Suchail , M. Valantin‐Morison , J.‐P. Sarthou , and J. Roger‐Estrade . 2013. “Nutritional State of the Pollen Beetle Parasitoid *Tersilochus heterocerus* Foraging in the Field.” BioControl 58: 17–26.

[pld370062-bib-0032] Samuelsson, G. , B. M. Sweeney , H. A. Matlick , and B. B. Prézelin . 1983. “Changes in Photosystem II Account for the Circadian Rhythm in Photosynthesis in *Gonyaulax polyedra* .” Plant Physiology 73: 329–331. 16663216 10.1104/pp.73.2.329 PMC1066461

[pld370062-bib-0033] Scialdone, A. , S. T. Mugford , D. Feike , et al. 2013. “Arabidopsis Plants Perform Arithmetic Division to Prevent Starvation at Night.” eLife 2: e00669. 23805380 10.7554/eLife.00669 PMC3691572

[pld370062-bib-0034] Silva, L. M. , L. P. Cruz , V. S. Pacheco , E. C. Machado , L. F. V. Purquerio , and R. V. Ribeiro . 2022. “Energetic Efficiency of Biomass Production is Affected by Photoperiod in Indoor Lettuce Cultivation.” Theoretical and Experimental Plant Physiology 34: 265–276.

[pld370062-bib-0035] Sorek, M. , Y. Z. Yacobi , M. Roopin , I. Berman‐Frank , and O. Levy . 2013. “Photosynthetic Circadian Rhythmicity Patterns of *Symbiodium*, the Coral Endosymbiotic Algae.” Proceedings of the Royal Society B 280: 20122942. 23554392 10.1098/rspb.2012.2942 PMC3619499

[pld370062-bib-0036] Stitt, M. , and S. C. Zeeman . 2012. “Starch Turnover: Pathways, Regulation and Role in Growth.” Current Opinion in Plant Biology 15: 282–292. 22541711 10.1016/j.pbi.2012.03.016

[pld370062-bib-0037] Sulpice, R. , A. Flis , A. A. Ivakov , et al. 2014. “ *Arabidopsis* coordinates the diurnal regulation of carbon allocation and growth across a wide range of photoperiods.” Molecular Plant 7: 137–155. 24121291 10.1093/mp/sst127

[pld370062-bib-0038] Webb, A. A. R. , and A. Satake . 2015. “Understanding Circadian Regulation of Carbohydrate Metabolism in Arabidopsis Using Mathematical Models.” Plant and Cell Physiology 56: 586–593. 25745029 10.1093/pcp/pcv033

[pld370062-bib-0039] Zhou, J. , J. Z. Wang , T. Hang , and P. P. Li . 2020. “Photosynthetic Characteristics and Growth Performance of Lettuce ( *Lactuca sativa* L.) Under Different Light/Dark Cycles in Mini Plant Factories.” Photosynthetica 58: 740–747.

