# Supplementary figures and images for: A Periodic 4‐h Extension of the Dark Period Did Not Cause Long‐Term Changes in the Circadian Regulation of Photosynthesis and Sugar Levels in Lettuces
Source: Plant Direct. 2025 Apr 21;9(4):e70062. doi: 10.1002/pld3.70062 (PMC12011635; doi:10.1002/pld3.70062)

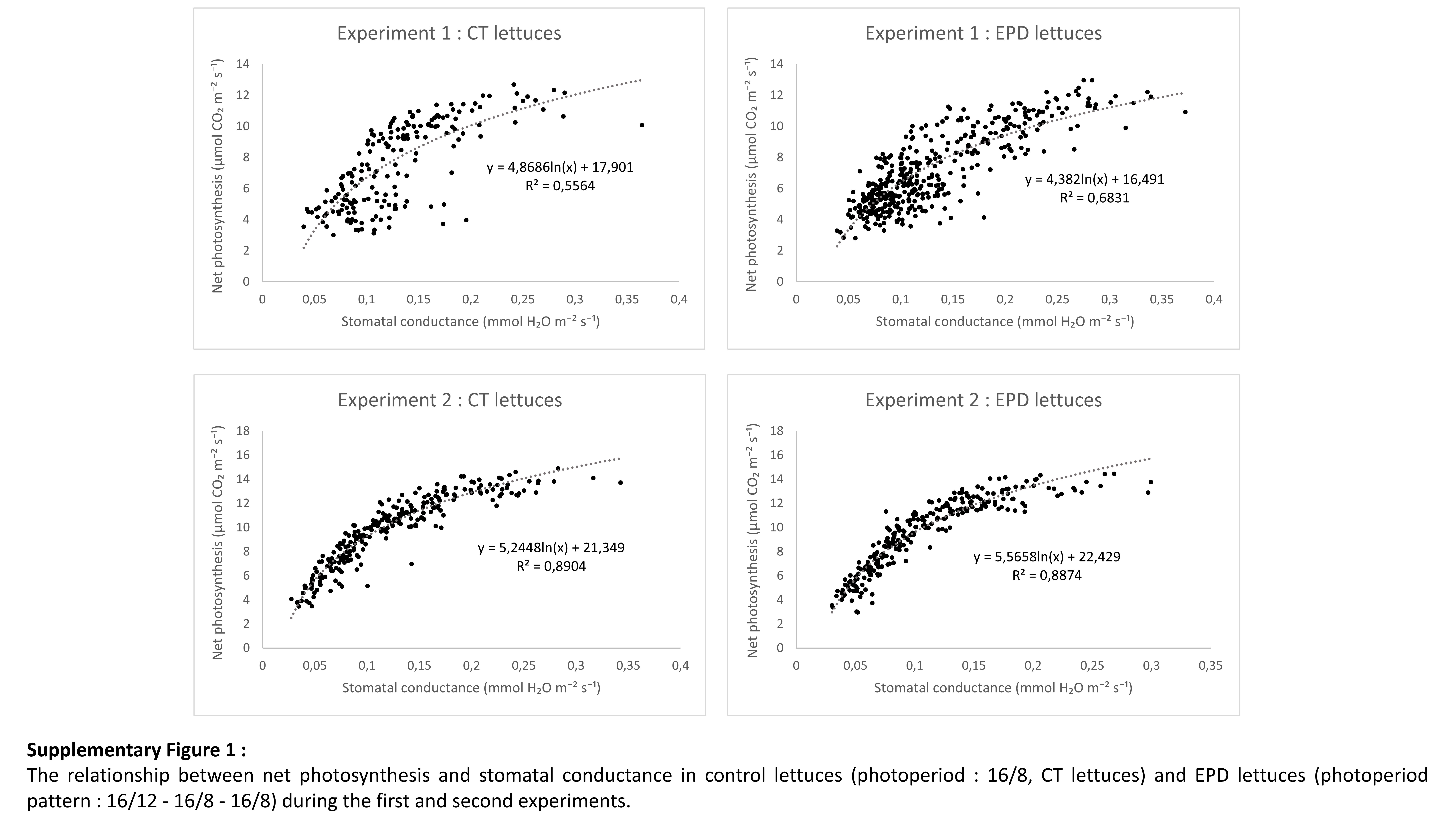

Supplement: Supplementary file 2 — Figure S1. The relationship between net photosynthesis and stomatal conductance in control lettuces (photoperiod: 16/8, CT lettuces) and EPD lettuces (photoperiod pattern: 16/12–16/8–16/8) during the first and second experiments. [file PLD3-9-e70062-s002.jpg]

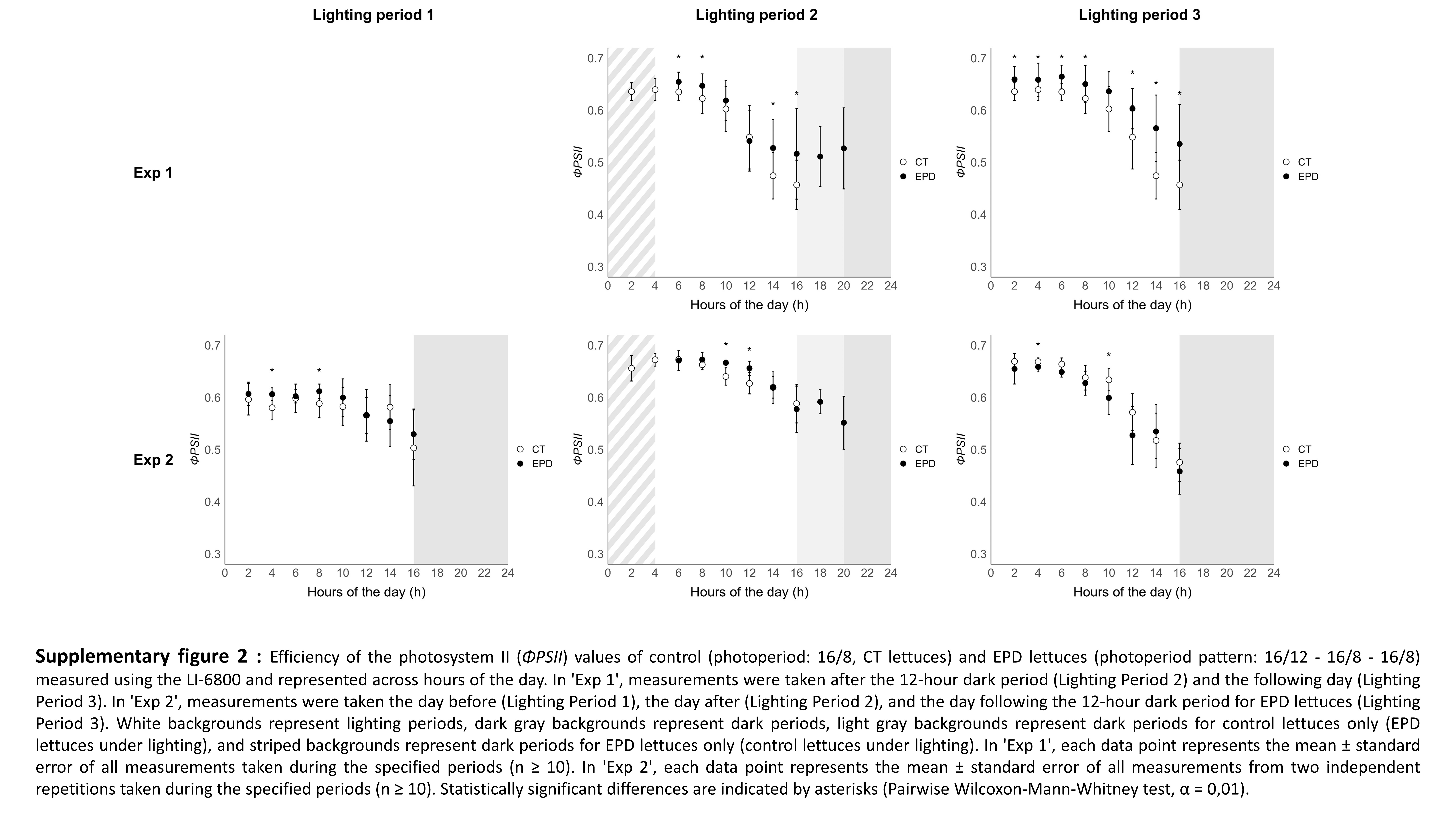

Supplement: Supplementary file 3 — Figure S2. Efficiency of the photosystem II (ΦPSII) values of control (photoperiod: 16/8, CT lettuces) and EPD lettuces (photoperiod pattern: 16/12–16/8–16/8) measured using the LI‐6800 and represented across hours of the day. In “Exp 1,” measurements were taken after the 12‐h dark period (Lighting Period 2) and the following day (Lighting Period 3). In “Exp 2,” measurements were taken the day before (Lighting Period 1), the day after (Lighting Period 2), and the day following the 12‐h dark period for EPD lettuces (Lighting Period 3). White backgrounds represent lighting periods, dark gray backgrounds represent dark periods, light gray backgrounds represent dark periods for control lettuces only (EPD lettuces under lighting), and striped backgrounds represent dark periods for EPD lettuces only (control lettuces under lighting). In “Exp 1,” each data point represents the mean ± standard error of all measurements taken during the specified periods (n ≥ 10). In “Exp 2,” each data point represents the mean ± standard error of all measurements from two independent repetitions taken during the specified periods (n ≥ 10). Statistically significant differences are indicated by asterisks (pairwise Wilcoxon–Mann–Whitney test, α = 0.01). [file PLD3-9-e70062-s001.jpg]

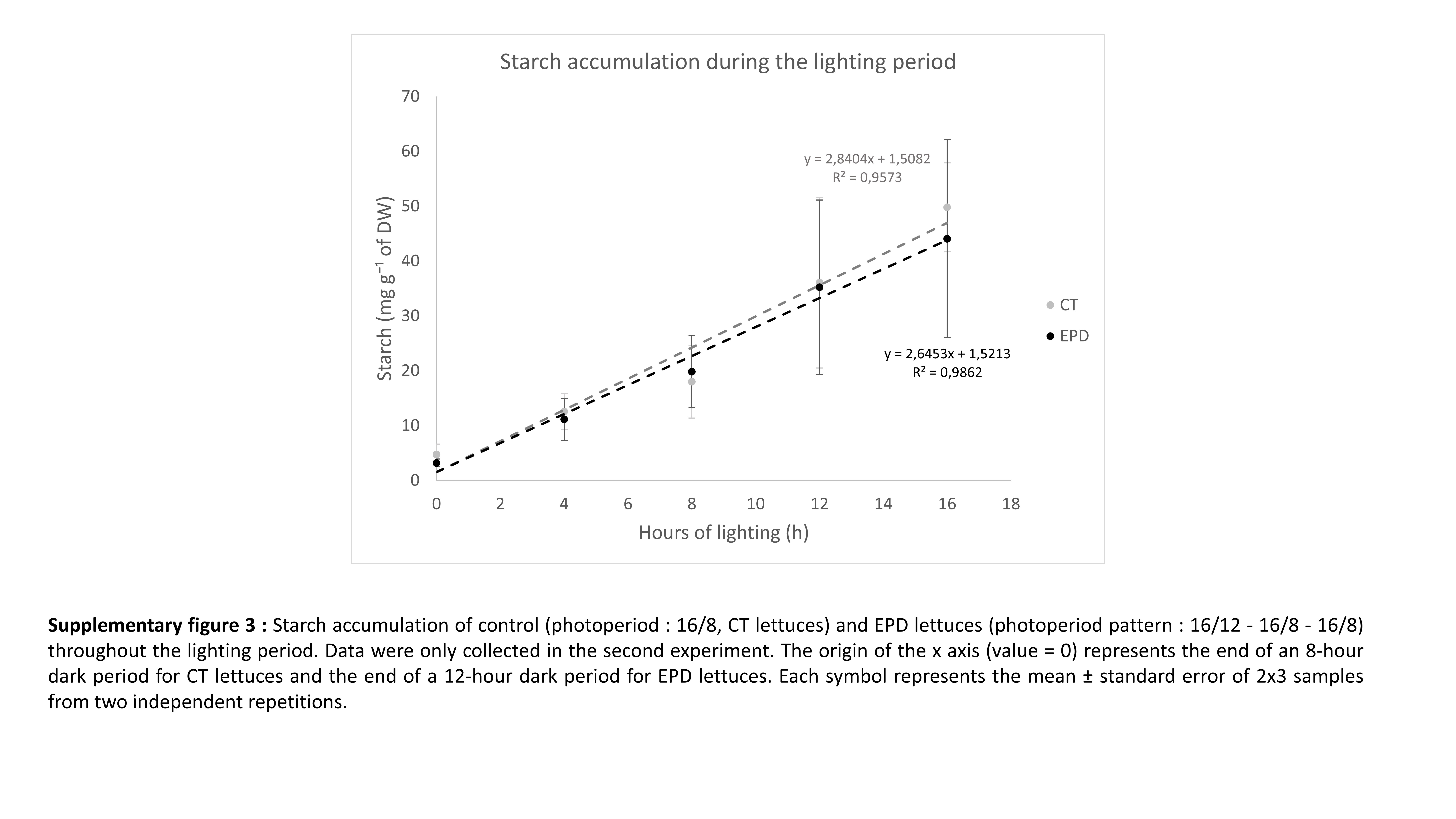

Supplement: Supplementary file 4 — Figure S3. Starch accumulation of control (photoperiod: 16/8, CT lettuces) and EPD lettuces (photoperiod pattern: 16/12–16/8–16/8) throughout the lighting period. Data were only collected in the second experiment. The origin of the x axis (value = 0) represents the end of an 8‐h dark period for CT lettuces and the end of a 12‐h dark period for EPD lettuces. Each symbol represents the mean ± standard error of 2 × 3 samples from two independent repetitions. [file PLD3-9-e70062-s003.jpg]
