# Supplementary material for: A Periodic 4‐h Extension of the Dark Period Did Not Cause Long‐Term Changes in the Circadian Regulation of Photosynthesis and Sugar Levels in Lettuces
Source: Plant Direct. 2025 Apr 21;9(4):e70062. doi: 10.1002/pld3.70062 (PMC12011635; doi:10.1002/pld3.70062)
Supplement: Supplementary file 1 — Data S1. Peer review. [file PLD3-9-e70062-s005.pdf]

Plant Direct
Open Access

A periodic 4-hour extension of the dark period did not cause long-term changes in the circadian regulation of photosynthesis and sugar levels in lettuces.

Cédric Dresch[1,2], Véronique Vidal[1], Séverine Suchail[3], Huguette Sallanon[1], Florence Charles[1,4], Vincent Truffault[2]

Decision Letter Round 1 :

December 6, 2023

Mr. Cédric DRESCH
Futura Gaïa
Agronomy
Rodilhan
France

RE: Photosynthesis is led by stomatal circadian rhythm and not by starch depletion after a long period of darkness

Dear Dr. DRESCH:

Thank you for submitting to Plant Direct. All required reviews have been returned and we have now finished our evaluation of your manuscript. In light of the reviewers' comments, further revisions are needed before the paper can be accepted for publication in Plant Direct. The main current limitations of the manuscript highlighted by the expert reviewers are:
a. Lack of a clear hypothesis or research question.
b. Lack of details about how the experiments were carried out and their reproducibility.
c. There is some redundancy in the writing and some of the data is shown multiple times.
d. The conclusions are not well supported by the data and will need to be adjusted.

Please view the editors' and reviewers' comments below and use their suggestions as a guide while you work on your revision.

When uploading the revised version of this article, please be sure to include the following:

-A word document that contains your response to the reviewers. You should respond to each reviewer comment and note the changes made to the manuscript. If you do not agree with a reviewer's comment and choose not to make a suggested revision, please explain why. Please try to provide as complete an

Plant Direct
Open Access
answer as possible to each reviewer's criticisms in the "response to reviewers" section.
-A tracked changes document
- A clean version of the latest version of the manuscript

To upload your revision, please click the link below.
https://plantdirect.msubmit.net/cgi-bin/main.plex?el=A7Lr4eG7A5oVn7I1A9ftdkYQAjcAP2r5EDsOZJnGvgZ

In order to provide as timely a service as possible, we ask that your revision is resubmitted within three months after receipt of this request. If an extension is needed, please send a request, along with a brief explanation, to the editorial office at plantdirect@wiley.com .

Thank you very much for giving us an opportunity to review your work. I look forward to receiving the next version.

Sincerely,
Eva Farre

-- Reviewer comments:
Reviewer #1:

Global comment

In the context of indoor farming and energy saving, Dresch et al. ask whether growing plants in non-regular circadian cycles significantly affects gas exchange and growth. The authors test this idea by submitting lettuce plants to extended nights through a photoperiodic pattern that does not match the circadian cycles (16 h light / 8 h dark for the control treatment CT vs. 16/12 - 16/8 - 16/8 for the EPD treatment). The pattern tested is very atypical and the rationale for this choice is not explicit, resulting in a scientific question that is unclear, an experimental design that is not easy to understand, and results that are difficult to interpret.

The data show that gas exchange is transiently impaired in the EPD treatment following the extended

night, because stomata start closing early in the photoperiod, likely due to circadian regulation: EPD appears simply shifted by 4h compared to CT on a Zeitgeber basis, just like stomatal dynamics still followed rhythm of the previous, normal day. On the following day, EPD recovers similar dynamics as CT on a Zeitgeber basis. This suggests that stomatal circadian rhythm is set at the time that light is switched on in the previous 24 h (but this point is not raised by the authors). Unfortunately, no gas exchange data is provided for the night periods and we do not know when stomata re-phase correctly. Moreover, alternative photoperiodic regimes (like always 16/12, or (12 h light / 12 h dark / 4 h light) - 16/8 - 16/8, or 4 h darkness randomly applied throughout a 16 h day...) are not tested, limiting our understanding of the (un)coupling between stomatal dynamics and the circadian clock.

The time-course of major carbohydrates is interesting, but not properly discussed. Glucose and fructose are always lower in EPD than in CT, yet the levels in both conditions are fairly constant and very high: 75-100 mg /gDW for Glc and 100-150 for Fru vs. a maximum of 100 for Suc and 40 for starch. Assuming the units are correct, this is in sharp contrast with Arabidopsis where the Glc + Fru is only about 10% of the maximum of Suc + starch (Gibon et al 2009 Plant J). This suggests that Glc and Fru act as long-term carbon buffers in lettuce. By contrast, sucrose and starch show marked diurnal oscillations. As expected, starch is depleted after the night extension, and recovers its normal turnover upon returning to control conditions. Suc dynamics is similar to that of starch, except in the EPD treatment where Suc dramatically increases at ZT48, i.e. the end of the normal night that follows the day when plants return to control conditions. Is such increase reproducible? From which carbon pool may it come from? Does it indicate that the clock misregulates sucrose metabolism, while it is able to correctly regulate stomatal movements at this stage? These points are not discussed.

The interrelations between starch/sugars, stomatal movements and the circadian clock are also not clearly presented. Key papers on mutual interactions are missing like Lascève et al 1997 PCE, Easlon & Richards 2009 PCE, Haydon et al 2013 Nature, Apelt et al 2017 Plant Phy, Vialet-Chabrand et al 2021 New Phytol, Westgeest et al 2023 Plant Cell, or the review of Flütsch & Santelia 2021 New Phytol. Instead, unconvincing argumentation is elaborated around the relationship between starch content and maximum photosynthetic capacity (lines 504-528).

Despite the fact that EPD plants had transiently impaired photosynthesis and had received less cumulated light at harvest, they do not show significant reduction in final plant weight, suggesting 5% light energy saving may be achieved without penalizing yield using the EPD pattern (yet yield was estimated on only 5 plants - see the major point below on reproducibility). No strong explanation is proposed except the idea that photosynthesis and pool of carbohydrates were not limiting for growth (which is not expected in the absence of stress). Alternatively, leaves of EPD plants may have expanded more than in CT during extended darkness (lower VPD), maybe compensating for reduced light availability through higher photosynthetic surface area? Unfortunately, leaf area per plant is not presented. Beyond this point, if the objective is to save light input by taking advantage of the circadian regulation, then the experimental strategy is hard to follow: here, light extinction was applied as a night prolongation, so that plants are light-deprivated in the subjective morning, i.e. when its photosynthetic

potential is at highest. Wouldn't it have been more relevant to shutdown light before the end of the subjective day, when stomata are already partly closed?

Major points

1. Reproducibility

Three types of variables are measured, with different issues regarding reproducibility:
• gas exchange (Pn, gs, fluorescence): it is written that "Measurements were taken manually with Anet and gsw values stability as recordable measurement indicators (3 to 5 minutes). Measurements were carried out on 10 plants (2 leaves per plant with the same phenological stage). During the measurements, plants were removed from the GiGrow® culture system and the potting soil of plant were rehydrated every hour to avoid water deficit.". Do the authors mean that they selected 10 plants that were regularly taken out and put back into the rotary system, or that these 10 plants were taken out for the whole photoperiod? If the latter, how was the light regime maintained? It should be clarified in the text. From the data, it could be inferred that each leaf was measured every about 2 h, so each plant about every 1 h. A control experiment should have been presented for making sure the transfer and/or regular leaf clamping with the Li6800 does not induce stomatal closure throughout the light period.
• carbohydrates (Glc, Fru, Suc, starch): the type of tissue sampled (whole aerial part? leaf pieces? only green parts of the lettuce?) is not indicated; according to the "Statistical analyses" section, n = 5 per time point, which seems acceptable.
• agronomical traits (FW, DW, SLA, LUE, WUE): according to the "Statistical analyses" section, n = 5, which is not sufficient for this type of highly integrated traits (statistical power is lacking here so it is not surprising that no difference could be detected).

Moreover, the experiment has not been independently replicated. This should have been done given that duration of the whole experiment is short (14 days nursery + 31 days rotary culture). This is crucial because i) statistical power may be lacking to detect significant differences in agronomical traits and ii) there are some unexpected results in the soluble sugars dataset (sustained decrease in glucose and fructose in EPD, and strange behaviour of sucrose at ZT48 for EPD). We need to be sure that it is not due to confusing effects (heterogeneity between the two rotary systems, treatments not harvested on the same day...).

Finally, the graphical layout and statistical analysis of gas exchange could be largely improved. It is almost impossible to see from the figure that "gsw values for CT lettuces increased from ZT0 to ZT2, peaking at a maximum mean value of 0.19 {plus minus} 0.07 moles H2O.m-2.s-1 between ZT3 and ZT4 (figure 2D). Subsequently, gsw values decreased to reach a stable mean value of 0.09 {plus minus} 0.03 moles H2O.m-2.s-1 from ZT11 to ZT16.". A smoothed line (loess or similar) with confidence interval for both treatments should be added. Colour and transparency may also be used for the points. Fig. 5 should be presented right after Fig. 2 (while reviewing the beginning of the results, I thought that the data of Fig. 2

should have been presented as done in Fig. 5, but I did not notice it until I read the discussion where it is first cited). Data of Fig. 3 could also be superimposed with that of Fig. 5 to produce a unique fit for all treatments that could then be compared to individual fits for each treatment.

2. Writing

The text is lengthy at many places, especially considering that the dataset presented here is restricted to one experiment and a few traits. The text should be much more concise. Moreover, the conclusions should not be overstated. Even in the title of the manuscript, the statement "and not by starch depletion" is not clearly established from the data presented here.

Minor comments

- Fig. 1C is misleading because treatments are aligned based on sum of daily light integrals, which progresses differently with real time between treatments, meaning that the interruptions in the axis (double vertical bar) that are used to "resynchronize" treatments graphically should have different lengths between treatments. A real-time representation would help here.

- line 34: "the circadian rhythm can be defined as a limiting factor for reducing energy consumption in indoor farming." and in the same vein line 556: "Interestingly, the exceptional disruptions of the circadian rhythm allowed to save 5% of lighting time compared to the control, which justifies that circadian rhythm has to be considered to reduce the energy consumption of indoor farming facilities while producing a marketable yield." These sentences are vague, and seem at odds with the conclusion that photosynthesis does not limit growth in these conditions.

- line 141: "A mutant-free study was carried out to avoid genes shutdown unintended side effects" (abstract and introduction) This sentence is unnecessary: on the one hand the authors don't have to justify themselves to work on a cultivar of a crop species, on the other hand it tends to be irritating for scientists working with mutants.

- line 158: "Plants were rotating at a speed of 50 minutes per revolution, which provides a centrifugal force of 1,33×10-5 Newton, considered as neglectable." according to which reference? any impact on starch statoliths?

- line 161: "density of 30 plants.m-2" per m2 of ground of per m2 flattened rotary culture?

- line 249: "Two leaves were dried in an oven at 80{degree sign}C for at least 3 days to calculate the dry weight." Unclear. Two leaves of each of the 5 plants? It was not determined on the whole plant like for the FW? It should be clarified.

- line 252: "2D plan" -> "2D plane"

- line 306: "interrelations" -> "correlations"

- line 388: "a decrease of light of approximately 5% was measured in EPD lettuces compared to the control". What is meant with "a decrease of light"? Does this number correspond to the reduction in the sum of DLIs over 31 d (= 1 - 584/610)? Was it measured or computed? It should be reformulated.

- line 457: "Indeed, circadian variations in photosystem II efficiency are difficult to describe because of their dependence on the leaf spot and the species studied (Allen, 2000; Rascher et al., 2001)." Unnecessary, this statement is true for basically every biological process...

- line 462: "In lettuces, we have demonstrated that the photosynthesis is regulated by the circadian rhythm of stomatal conductance." I would not say demonstrated, no experiment has been performed under continuous light, etc...

- line 467: "After an exceptional darkness period of 12 hours, starch residual content of lettuces was equal to 12% of the initial stock, compared to 37% for the control after 8 hours of darkness". Unclear, not in line with the previous sentence. Which point was considered as the initial stock? The argumentation on starch degradation rate is also very unclear: how starch degradation rate can be estimated during the night extension while no data is available at the end of the previous day? I would simply say that starch depletion is observed after a prolongation of the night, while normal starch content is recovered when photoperiodism comes back to its entrainment regime.

- line 502: "these results suggest that starch is the best candidate as to be indicator of the circadian rhythm amongst the studied sugars." sugars -> carbohydrates; this sentence is overstated, not only there is no apparent link between starch and the clock in the data presented here, but also sucrose looks probably more interesting (but its dynamics has to be independently replicated).

Reviewer #2:

Comments on 'Photosynthesis is led by stomatal circadian rhythm and not by starch depletion after a long 2 period of darkness'.
General Comments
The manuscript focuses on Lettuce response to an extended night period. Authors looked into photosynthetic changes during the light period right after an extended night and compared them to a control treatment of constant day/night periods. They complement this response with several morphological variables measured at the end of the growth cycle. They found slight changes in net CO2 assimilation, no changes in stomatal conductance, and no differences between morphological variables. In this context they argue that the circadian rhythm controls stomatal conductance and the latter

controls net assimilation. The literature on this topic is mixed, and many suggest that changes are driven by carbohydrate synthesis. The authors also suggest that this extended night period could increase the energy efficiency of the system, without affecting yields. I am positive the topic would be of interest for the readers of Plant Direct, however, the manuscript is not yet ready for publication and major changes need to be addressed.
Overall, the manuscript reads with ease, but it presents majors flaws that need to be addressed.
The introduction is merely descriptive and does a poor job guiding the reader to a knowledge gap. Authors spend most of the introduction describing already-known processes and barely comment on the scientific question they are trying to answer. In addition, multiple sentences read disconnected and lack proper reference support. Also, the introduction does not state any clear objectives or hypothesis, which makes reviewing the manuscript extremely hard.
The methods section does not provide enough detail on the experimental design, the growth system, and the methodology and analysis of some variables. A better description of the rotary culture system is much needed, what is the purpose of the rotation? is it rotating vertically or horizontally? Where is the light source? What is the CO2 concentration? was it controlled?
And, most importantly, how many were used in the experiment? This brings a very important point, the number of replicates is not very clear, based on comments in the results section, it seems it only one per treatment, is this right? If so, it could be a major flaw as it would not capture the variability. Authors must discuss this topic and how they overcome this limitation.
More detail is also need in how the Licor measurements were collected and analyzed. The authors said that photosynthesis was measured continuously for 16hr, and that measurements were taken manually on 10 plants. Saying 'continuously' could mean that they did not unclamp the leaf and were measuring using the Dynamic Assimilation Technique. If that is the case, and if they measured 10 plants, does it mean you used 20 licors? How many instruments did you use?
Finally, what was the statistical analysis used for photosynthesis data?
The Results section is also very descriptive and tends to repeat the data. I would encourage the authors to be more clear when presenting percentage changes, and to add p-values when comparing treatments. Also, Figures 2, 3 and 5 have serious issues. First, do not repeat data, there is no reason to present data of each treatment separately (A, B) and then combined (C), just present C. Also figure E should be split. Each variable should be its own plot.
Also is it possible to add some type of trend for each treatment, it would make it much easier to see the patterns. Also, what does each point represent? You mentioned there are 10 plants and two leaves per plant, is data averaged in some way? Could they be averaged over reps? As presented, the data seem noisy which makes it hard to see the trends between treatments.
In my opinion the discussion does not provide enough support to the main idea of the manuscript that the circadian rhythm control stomatal conductance and the later controls photosynthesis. References are scarce, a bit outdated, and mainly on Arabidopsis. I understand the value of a model species, but work done in lettuce or other leafy greens would be more informative.
Another important point is that a big piece of evidence for support the circadian control on stomatal conductance and assimilation is a ~ 6% decrease in assimilation. I think this has three limitations; first, 6% is not a large difference mainly considering the large variability in your dataset, second, the average

may not be the most indicative parameter, instead total daily assimilation could provide a better description of the patterns, and third, you also report and increase in A_net in the early hours of the light period, but it is not incorporated in the discussion.
Another aspect that is missing in the discussion is whether the extended night period is enough to cause a change in the circadian rhythm. It could be that plants coming out of the 12hr night period did not acclimate to the change and they responded as the control.
These limitations make it very hard to conceptually review the manuscript and must be addressed. I am also attaching a pdf with in-line comments in the manuscript and figures. Some may be more relevant than others, but please have a look at them.

Decision Letter Round 2 :

July 10, 2024

Mr. Cédric DRESCH
Futura Gaïa
Agronomy
Rodilhan
France

RE: An exceptional increase in the darkness period induces a phase shift in photosynthesis of indoor-grown lettuces

Dear Dr. DRESCH:

Thank you for submitting to Plant Direct. All required reviews have been returned and we have now finished our evaluation of your manuscript. In light of the reviewers' and editor's comments, further revisions are needed before the paper can be accepted for publication in Plant Direct.

Although the writing and presentation of the manuscript has been improved, this current version does not provide enough evidence that the experiments presented are reproducible, which is essential for publication in Plant Direct. All the data used for the analysis needs to be shown or if data from other studies is used a reference needs to be provided. If you do have additional repeated experiments, as your seem to indicate in the rebuttal letter, it will be important to incorporate them in the manuscript. I would also suggest going over the comments and suggestions of the reviewers for revising the interpretation of the results of the experiments. Please view reviewers' comments are below.

Plant Direct
Open Access

When uploading the revised version of this article, please be sure to include the following:

-A word document that contains your response to the reviewers. You should respond to each reviewer comment and note the changes made to the manuscript. If you do not agree with a reviewer's comment and choose not to make a suggested revision, please explain why. Please try to provide as complete an answer as possible to each reviewer's criticisms in the "response to reviewers" section.
-A tracked changes document
- A clean version of the latest version of the manuscript

To upload your revision, please click the link below.
https://plantdirect.msubmit.net/cgi-bin/main.plex?el=A5Lr6eG5B2oVn6I6A9ftdkYQAjcAP2r5EDsOZJnGvgZ

In order to provide as timely a service as possible, we ask that your revision is resubmitted within three months after receipt of this request. If an extension is needed, please send a request, along with a brief explanation, to the editorial office at plantdirect@wiley.com .

Thank you very much for giving us an opportunity to review your work. I look forward to receiving the next version.

Sincerely,
Eva Farre

-- Reviewer comments:
Reviewer #1:

I have reviewed again the manuscript by Dresch et al.

The major issue of reproducibility remains. There is only one independent experiment for each treatment (control and EPD), and each treatment was assessed in a separate experiment. Can we detect a yield reduction with more statistical power? Are Glc and Fru contents always lower in the EPD treatment (potentially underlying the preservation of yield in spite of the decrease in photosynthesis)? These questions will remain if only one experiment is presented. It is claimed in the rebuttal that "In fact, we have repeated the whole experiment and yields have always been the same" but data are not shown or even mentioned in the manuscript. Alternatively, other light scenarios may have been explored to

challenge the proposed model - but the findings are based on a single experiment.

The text has been clarified and made more concise as recommended. While I appreciate these efforts, there is still a large imbalance between the results (about 700 words) and the discussion (about 1800 words). Some parts are still hard to follow (e.g. lines 54-67, 369-384). English needs revision as well.

The figures have been improved, yet I regret that gas exchange measurements were binned into 4 discrete periods because it blurs the actual temporal patterns - making the phase shift much less perceptible. A similar layout like Fig. 5D and E would have been more appropriate (with statistics on binned data possibly added in Supp Data).

As mentioned in your rebuttal, the Suc data have been corrected. Comparing the data between the previous and actual versions, it seems that two points have been switched (40 and 48h for EPD lettuces). Such a switch would have deserved an explanation. It is a pity that the mistake was not detected before, given the important implications for the interpretation.

Lines 327-352: the discussion about potential adjustment of starch degradation rate does not make sense. Gibon et al. (2004) showed that starch degradation rate slows down when the night is extended due to an anticipated start of the night. In your manuscript, night is extended due to a delay in the start of the next day, so there is no way for the plant to anticipate this change - resulting in a mismatch between stomatal opening and light unavailability. Moreover, the starch degradation rates mentioned here are computed based on data not shown.

Reviewer #2:

I appreciate the authors for taking care of my general comments. The manuscript has improved in this round of revisions.
That said, I think the manuscript still has two main pitfalls. First, the introduction does not do a good job introducing the knowledge gap. Authors spend a lot of space describing processes that are already well known and understood but fail to introduce how these processes interact among each other in the context of their research. To be clear, more details need to be provided about the interaction between photoperiod, circadian rhythm, photosynthesis (CO_2 assimilation and stomatal conductance) and carbohydrates.
For the second point, I would encourage the authors to think of the results in terms of the source-sink balance of the leaf. See below my comments on how the initial increase in assimilation could be driven by the depleted reserves of the extended night, and it may only take a few hours for the plant to restore the carbohydrate balance. As is, it is very biased toward the changes in stomatal conductance, but there is not much support from previous studies or from their own results.
Also see below the detailed comments. Many of them were already in the pdf of the previous version. I

hope having them here helps the authors to include them.
L 1: Phase shift is ambiguous
L 47: The intro is missing key points of the research and add too much detail in already established knowledge. I suggest adding more detail into how photoperiod affects photosynthesis, whether these effects are driven by stomatal conductance, circadian cycle and/or source sink balance of carbohydrates. After reading your discussion, these the three main topics you discussed and are not introduced.
Also, it's known that plants adapt to constat changes in the photoperiod, but what is it known about plants adapting to more complex cycles, such as the one in your study? This is very important and should be included in the intro.
L49 - 85% of what?
L50 - Don't need this, you aren't going to talk about it later.
L52 - I like how you reshaped this paragraph. I think it would be important to highlight the knowledge gap you are trying to fill. A sentence like this makes it seem that there is no knowledge gap and there is no need to do you study.
L55 Add hyphen.
L55 - instead of multiplied by two use "doubled"
L63 - Not sure what you are trying to say.
L 69 - change for 'and'
L 70 - I understand the relevance of photosynthesis to this research but there is absolutely no point in describing such a well-known process here. I mentioned this in the previous round and the problem persists. Instead of spending half a page in photosynthesis, you should introduce how photosynthesis could be affected by circadian and photoperiod changes.
L 86 - Same as with the previous paragraph, this is merely descriptive and is not related to the research question.
L105 - Two points here:
1- Does no one done any research on extending the night period? This the research you should be mentioning in the intro.
2 - This needs further clarification, as it reads it's a bit confusing how is extending the night period an energy saving opportunity. If you are keeping the lights on for the same time then the energy consumption would be equal. The key point (I think) is that plants grew for the same number of days.
L 113 Somewhere you must explain how treatments were implemented. Based on the response to the reviewers, you mentioned that you did one treatment and then the other. You must explain this and discuss that it doesn't affect the measure. You run multiple runs of this, you should also present the data
L152 It would be very useful if you could indicate in this table when the different samples took place. Also, based on the figure is not clear to me where the energy savings are coming from. Despite EPD plants having a total of two longer nights, it seems these extra 8 hours are not removed from the total length of the period. Then it seems that the EPD would end their growing period later. I made a point in the objective about this. This should be explained in further detail and represented accordingly.
L 160 how could be consistently equal if they are not controlled?
L164 All figures should also include the days after transplant.
L168 just to be clear, you have an n = 1. all plants are subsamples of the single treatment. This must be

discussed in the discussion or methods. Change n in your figures and say the number of plants.
L 225 any time you mentioned significant, you must say the pvalue at which you consider it. Also, provide the p-values of the comparisons when you mentioned them on the text.
L226 Also, mention the functions and packages you used.
L 226 I don't think this is the right way to refer to R studio. citation() in Rstudio gives:
R Core Team (2022). R: A language and environment for statistical computing. R Foundation for Statistical. Computing, Vienna, Austria. URL https://www.R-project.org/.
L 232 Addressing morphology from a picture is challenging. As the other reviewer mentioned, leaf area would be a better index.
L236 here and for the rest of the manuscript; avoid single-sentence paragraph.
L 239 It seems a bit odd to represent the time with such a large interval. Did it take you 4 hours to measure all plants? if so, you may need to do some corrections by leaf. If not, present the average of the times
L 242 After reading the discussion, this sentence seems to be very important. You must be more clear about how this 6.2% was calculated. Was it the average during the day? I think there is also a lot of value in analyzing this data by each time point. Based on the graphs it seems that it only takes the leaf the first hours of the day to restore the imbalance.
L 260 You only have data for the beginning and end of the light phases, then you can't say what happened during the rest of the photoperiod. Saying that is stables is an overstatement, and can't be supported with your measurements. It could be that they go up in concentration and then return to the initial values
L 263 what's the pvalue of this test, I see that they tend to increase in the graph but the deviation is pretty large. To be honest, the lack of p-values is starting to make me doubt about the validity of your tests. Please add pvalues and more detail in the stat section
L 288 Daily usually refers to the total amount of net assimilation accumulated during the day. Please use a different term.
L289 Need references to support this.
L 290 The link between anet and gsw seems to be a key finding of you research but it is poorly described. please expand on the reasoning and how your data supports this. I personally don't think you have the data to support that is gsw that drives the decline in Anet, and would like to read more about your explanation.
L 294 You don't need to present the pvalue of another research. The R2 could be more informative. That said, you MUST report the pvalue of your tests.
L 297 why do you define stomatal conductance now? do it the first time you mentioned it.
L298 This is a very important point of your manuscript. I would suggest supporting it with other studies. Also, I would include part of the main points in the introduction.
One aspect that is missing with this explanation is the rates of Anet being higher during the first measurement. Your data show a clear correspondence between increased gsw and Anet but your hypothesis doesn't explain the increase in Anet.
If we look at this trend from source-sink perspective, increased A net could be driven by the lower sucrose levels. Unfortunately, you don't have a diurnal course of carbohydrates. Based only on Anet, and

assuming a direct correspondence with sucrose. It could be that it only takes ~4hours for the EPD plants to potentially replete the reserves to CT levels, and from there on, the source-sink balance is restored and photosynthesis goes at the same rate.
L 303 See my previous comment, I think these minor differences could be what EPD plants need to restore the source sink balance.
L 328 does 'which' refer to photosynthesis or the circadian rhythm
L 329 had
L 336 why not? the increased Anet at the beginning of the day could be what drove the increased starch accumulation.
L 339 Could
L 341 How could this translate to your study? in your case the plants experience the increased light period 1 every 3 days, how would the plant know that it's experiencing an extended darkness period?
L 347 but you just said that it was adjusted in arabidopsis?
L 357 what this means?
L 359 your study? or the one referenced? please be clear
L 362 in which species?
L 373 why are you making the decrease in sugars being NOT correlated with Photosynthesis? From a source sink perspective, it should be the opposite. A
L 375 but it was higher in the first time of the day??? It may be all the plant needs to readjust
L 376 This needs to be further discussed. There is lot of literature showing the opposite, in fact trio-phosphate utilization is one the main limitations of photosynthesis
L 381 Which other tissues? And how this relates to your study
L 385 Your data doesn't support this statement. You saw that the without a change in starch there was not change in yield, but you didn't do any experiment to test show this causation.
L 387 This paragraph is a bit confusing, it starts mentioning how assimilation doesn't change and then talks about fresh weight. I think a big missing component in the intro is how changes in photoperiod interact with photosynthesis and ultimately biomass. Something about the effects of sucrose and starch and the imbalance of the source-sink system may affect biomass.
L 388 cite one of your figures/tables,
L 393 is there a missing word here??
L 404 This reasoning is missing the fact that both the 6% decline in Anet and the decreased in carbohydrates are diurnal averages after the change photoperiod. It could well be that the EPD plants readjust their source sink balance within the first hours of the day and that is enough to compensate for the declines in carbs.
Another aspect that is missing in here and in the discussion is the idea of 'storage carbohydrate' the fact that glucose and fructose are constant at the beginning and end of the day may say that this are produced and consumed during the day. Sucrose and starch change increase during the day suggesting this are the storage carbohydrates. These are more relevant to the source-sink balance of the leaf. In both cases they are further depleted right after the extended night but replenished to control levels within one day. it seems that within the first few hours of the day (the first timepoint) leaves are able to replenish the reserves and then they returned to control dynamics.

L 413 This just repeats the previous sentence. It would be good to add more support,
L 415 Daily photosynthesis is commonly used to refer to the integration of net assimilation over the day. I suggested this on my previous review. Please use a different term.
L 423 Where does this 5% comes from? It should be explained somewhere or if the math is simple added between brackets here
L 474 This table is very interesting, and it's barely mentioned in the results. Do you have any hypothesis of why EPD plants are almost 15% more efficient than control?
Good Job!! The manuscript has improved from the previous version. I think adding these will notably increase the quality

Decision Letter Round 3 :

December 11, 2024

Dr. Cédric DRESCH
Futura Gaïa
Agronomy
Rodilhan
France

RE: A periodic 4-hour extension of the dark period did not cause long-term changes in the circadian regulation of photosynthesis and sugar levels in lettuces.

Dear Dr. DRESCH:

Thank you for submitting to Plant Direct. We have now reviewed your revised manuscript and there are further revisions that are needed before the paper can be accepted for publication in Plant Direct.

Please view the editors' comments below and use their suggestions as a guide while you work on your revision.

When uploading the revised version of this article, please be sure to include the following:

-A word document that contains your response to the reviewers. You should respond to each reviewer comment and note the changes made to the manuscript. If you do not agree with a reviewer's comment and choose not to make a suggested revision, please explain why. Please try to provide as complete an answer as possible to each reviewer's criticisms.

Plant Direct
Open Access

-A tracked changes document with each change highlighted
- A clean version of the latest version of the manuscript

To upload your revision, please click the link below.
https://plantdirect.msubmit.net/cgi-bin/main.plex?el=A2Lr2eG2C4oVn6I2A9ftdkYQAjcAP2r5EDsOZJnGvgZ

In order to provide as timely a service as possible, we ask that your revision is resubmitted within three months after receipt of this request. If an extension is needed, please send a request, along with a brief explanation, to the editorial office at plantdirect@wiley.com .

Please note that, in addition to publishing reviewer comments, the author's responses to review comments will also be published alongside the final version of the paper. If you would not like the author's responses to be published, please contact the editorial office at plantdirect@wiley.com .

Thank you very much for giving us an opportunity to review your work. I look forward to receiving the next version.

Sincerely,

Eva Farre

Editor, Plant Direct

-- Editor comments:

Thank you for adding the data for an additional experiment and incorporating the suggestions of the two reviewers.

The main issue that needs to be addressed are the apparent discrepancies on how these experiments were performed as shown in the depictions of dark and light periods in Figures 1B and 4. Here are a couple of more comments and suggestions that should help further improve the manuscript.

Line 196: The phrase 'repeated period of darkness of 12 hours', is slightly confusing, since the key change

is the elongation of the night by 4 h which causes a phase delay. More informative would be "... by repeated elongations of the night period'. There seem to be some discrepancies between Figure 1B and Figure 4 on how the treatments were done, please see my comment below.

Line 200: should be ' differences in fresh weight and dry weight between the two conditions were only detected...'. Since these differences were not reproducible between experiments, it would be more informative to the reader to write that not reproducible differences in biomass were detected between the two treatments.

It would be useful to indicate in Figure 1B what the authors define as 'Lighting period 1' and 'Lighting period 2' and 'Lighting period 3'.

The 3rd, or 4th panel in Figures 2A/B and 3A/B, are not very informative. They show the 'CT' treatments as if they were measured in the dark (although that is clarified in the legend). It is rather confusing. Based on Figure 1 than the EPD plants would have been subjected to a phase shift, but that is not how Figure 4 shading is depicted.

Here again, for the data shown in Figures 2 and 3, Experiments 1 and 2 do not show consistent differences between CT and EPD treatments. Therefore it is only distracting to the reader describe in detail differences that are not consistent between experiments.

It is unclear what which data is shown in each panel. The EPD LN treatments have only one time point. It might be clearer to indicate the lighting period as for figures 2 and 3. There are again only very few, if any, differences between the experiments.

Line 263: The authors write that the EPD treatment "affected the daily trend of net photosynthesis". I do not think there are any consistent trends between Experiment 1 and Experiment 2 (Figures 2 and 3). I also do not think the data shown provides any evidence for or against circadian control.

Line 281: The authors write that 'due to the extension of the night, only 4 hours of maximal net photosynthesis remained'. I am not sure what is meant by this. According to Figure 1B, the extension of the night leads to a phase shift, the next light period, after the extended dark, is also 16 h. However, I see that that is not the case in the Figure 4, in which the light period after the extended dark is only of 12 h. This needs to be clarified. I cannot see any consistent decrease in carbon assimilation in EPN conditions across both experiments in any of the lighting periods. As the reviewers indicated about previous versions of the manuscript is unclear how the decrease in the daily photosynthesis of EPD lettuces was calculated.

Line 297. A main difference of the described experiments and other studies is that in this one the plants are treated to a 4 hour 'jet lag' every 3 days. Other experiments grew plants on different T-cycles constantly. So it seems that a more 'sporadic' phase change does not affect the plants strongly.

Other comments:
Numbers: decimals are indicate with "." not ","
units: please don't use "." such as umol.m (=micromol per meter), just use a space or "x", but please be consistent, don't use space and 'x'.

Line 151: should be 'light' not 'lighting'
Line 156: explain HPAEC abbreviation

Line 178: please provide full protocol or reference. This protocol does not allow reproducibility.

It is unclear how fresh and dry weight were measured. Were these assays distructive? i.e. were whole plants harvested?. In Line 189 it said that only 2 leaves were dried, is that sufficient to estimate the dry weight of the whole plant?

Decision Letter Round 4 :

January 4, 2025
Dr. Cédric DRESCH
Futura Gaïa
Agronomy
Rodilhan
France

MSID: 2023-01343-TW123
MS TITLE: A periodic 4-hour extension of the dark period did not cause long-term changes in the circadian regulation of photosynthesis and sugar levels in lettuces.

Dear Dr. Cédric DRESCH:

I am pleased to inform you that your manuscript "A periodic 4-hour extension of the dark period did not cause long-term changes in the circadian regulation of photosynthesis and sugar levels in lettuces." has been accepted for publication in Plant Direct.

Your article will appear online in the next available issue of Plant Direct. To ensure your article gets published as quickly as possible, please pay attention to the steps detailed below. We have found that most of the delays happen at this stage, especially at the payment stage, so please respond as quickly as

possible when prompted.

License Agreement: Once your article has been accepted it will move to Production and undergo admin and file checking - you may receive an email with any queries we have at this stage. When all required items are received by the publisher and queries resolved, the corresponding author will receive an email from Wiley's Author Services system which will ask them to log on at https://authorservices.wiley.com/bauthor and will present them with the appropriate license for completion. Your article cannot be published until both the signed license agreement and payment of the article fee have been received.

Payment of the Open Access Article Publication Fee: All articles published in Plant Direct are fully open access: immediately and freely available to read, download and share. Plant Direct charges a publication fee to cover the publication costs. The corresponding author for this manuscript should have already received a quote from the payments team (cs-openaccess@wiley.com) with the estimated article publication fee; please email cs-openaccess@wiley.com if this has not been received. The corresponding author should log on to the Wiley Author Services site, where the publication fee can be paid by credit card or an invoice. Pro Forma can also be requested. Payment of the publication charge must be received before the article will be published online.

Proofs: You will have the opportunity to look over your paper once more when you receive the author proofs for your article. The proofs will be with you in approximately two weeks. Please note that, in addition to publishing reviewer comments, the author's responses to review comments will also be published alongside the final version of the paper. If you would not like the author's responses to be published, please contact the editorial office at plantdirect@wiley.com .

Promotion of your article: You can help your research get the attention it deserves! Check out Wiley's free Promotion Guide for best-practice recommendations for promoting your work at www.wileyauthors.com/eeo/guide. And learn more about Wiley Editing Services which offers professional video, design, and writing services to create shareable video abstracts, infographics, conference posters, lay summaries, and research news stories for your research at www.wileyauthors.com/eeo/promotion.

Thank you again for your contribution to Plant Direct. If you have any questions, feel free to contact the editorial office at plantdirect@wiley.com .

Sincerely,

Eva Farre

Gustavo MacIntosh

Plant Direct
Open Access

Editor, Plant Direct

Response to Reviewer Round 1 :

Dear Reviewers,

We are grateful for your feedback on our manuscript. Your critiques have significantly contributed to refining our work, and we have addressed each of your comments to enhance the clarity and robustness of our study. Enclosed, you will find detailed responses to each point raised (*cf.* comments), along with corresponding revisions in the manuscript.

The authors

Reviewer #1:

Global comment

In the context of indoor farming and energy saving, Dresch et al. ask whether growing plants in non-regular circadian cycles significantly affects gas exchange and growth. The authors test this idea by submitting lettuce plants to extended nights through a photoperiodic pattern that does not match the circadian cycles (16 h light / 8 h dark for the control treatment CT vs. 16/12 - 16/8 - 16/8 for the EPD treatment). The pattern tested is very atypical and the rationale for this choice is not explicit, resulting in a scientific question that is unclear, an experimental design that is not easy to understand, and results that are difficult to interpret. The data show that gas exchange is transiently impaired in the EPD treatment following the extended night, because stomata start closing early in the photoperiod, likely due to circadian regulation: EPD appears simply shifted by 4h compared to CT on a Zeitgeber basis, just like stomatal dynamics still followed rhythm of the previous, normal day. On the following day, EPD recovers similar dynamics as CT on a Zeitgeber basis. This suggests that stomatal circadian rhythm is set at the time that light is switched on in the previous 24 h (but this point is not raised by the authors). Unfortunately, no gas exchange data is provided for the night periods and we do not know when stomata re-phase correctly. Moreover, alternative photoperiodic regimes (like always 16/12, or (12 h light / 12 h dark / 4 h light) - 16/8 - 16/8, or 4 h darkness randomly applied throughout a 16 h day...) are not tested, limiting our understanding of the (un)coupling between stomatal dynamics and the circadian clock.

Commented [CD1]: We have clarified the scientific question by reformulating the introduction.

The time-course of major carbohydrates is interesting, but not properly discussed. Glucose and fructose are always lower in EPD than in CT, yet the levels in both conditions are fairly constant and very high: 75-100 mg /gDW for Glc and 100-150 for Fru vs. a maximum of 100 for Suc and 40 for starch. Assuming the

units are correct, this is in sharp contrast with Arabidopsis where the Glc + Fru is only about 10% of the maximum of Suc + starch (Gibon et al 2009 Plant J). This suggests that Glc and Fru act as long-term carbon buffers in lettuce. By contrast, sucrose and starch show marked diurnal oscillations. As expected, starch is depleted after the night extension, and recovers its normal turnover upon returning to control conditions. Suc dynamics is similar to that of starch, except in the EPD treatment where Suc dramatically increases at ZT48, i.e. the end of the normal night that follows the day when plants return to control conditions. Is such increase reproducible? From which carbon pool may it come from? Does it indicate that the clock misregulates sucrose metabolism, while it is able to correctly regulate stomatal movements at this stage? These points are not discussed.

Commented [CD2]: Sucrose data have been corrected

Commented [CD3]: Added to the discussion

Commented [CD4]: We agree with the reviewer and have adapted the manuscript accordingly.

The interrelations between starch/sugars, stomatal movements and the circadian clock are also not clearly presented. Key papers on mutual interactions are missing like Lascève et al 1997 PCE, Easlon & Richards 2009 PCE, Haydon et al 2013 Nature, Apelt et al 2017 Plant Phy, Vialet-Chabrand et al 2021 New Phytol, Westgeest et al 2023 Plant Cell, or the review of Flütsch & Santelia 2021 New Phytol. Instead, unconvincing argumentation is elaborated around the relationship between starch content and maximum photosynthetic capacity (lines 504-528).

Commented [CD5]: This part has been removed, and the literature has been added.

Despite the fact that EPD plants had transiently impaired photosynthesis and had received less cumulated light at harvest, they do not show significant reduction in final plant weight, suggesting 5% light energy saving may be achieved without penalizing yield using the EPD pattern (yet yield was estimated on only 5 plants - see the major point below on reproducibility). No strong explanation is proposed except the idea that photosynthesis and pool of carbohydrates were not limiting for growth (which is not expected in the absence of stress). Alternatively, leaves of EPD plants may have expanded more than in CT during extended darkness (lower VPD), maybe compensating for reduced light availability through higher photosynthetic surface area? Unfortunately, leaf area per plant is not presented. Beyond this point, if the objective is to save light input by taking advantage of the circadian regulation, then the experimental strategy is hard to follow: here, light extinction was applied as a night prolongation, so that plants are light-deprivated in the subjective morning, i.e. when its photosynthetic potential is at highest. Wouldn't it have been more relevant to shutdown light before the end of the subjective day, when stomata are already partly closed?

Commented [CD6]: We ended with the same conclusion, we are actually working on this topic of removing lighting hours that are the less efficient for photosynthetic activity.
We have clarified the outputs of the conclusion.

Major points

1. Reproducibility

Three types of variables are measured, with different issues regarding reproducibility:

• gas exchange (Pn, gs, fluorescence): it is written that "Measurements were taken manually with Anet and gsw values stability as recordable measurement indicators (3 to 5 minutes). Measurements were carried out on 10 plants (2 leaves per plant with the same phenological stage). During the

measurements, plants were removed from the GiGrow® culture system and the potting soil of plant were rehydrated every hour to avoid water deficit.". Do the authors mean that they selected 10 plants that were regularly taken out and put back into the rotary system, or that these 10 plants were taken out for the whole photoperiod? If the latter, how was the light regime maintained? It should be clarified in the text. From the data, it could be inferred that each leaf was measured every about 2 h, so each plant about every 1 h. A control experiment should have been presented for making sure the transfer and/or regular leaf clamping with the Li6800 does not induce stomatal closure throughout the light period.

- carbohydrates (Glc, Fru, Suc, starch): the type of tissue sampled (whole aerial part? leaf pieces? only green parts of the lettuce?) is not indicated; according to the "Statistical analyses" section, n = 5 per time point, which seems acceptable.
- agronomical traits (FW, DW, SLA, LUE, WUE): according to the "Statistical analyses" section, n = 5, which is not sufficient for this type of highly integrated traits (statistical power is lacking here so it is not surprising that no difference could be detected).

Moreover, the experiment has not been independently replicated. This should have been done given that duration of the whole experiment is short (14 days nursery + 31 days rotary culture). This is crucial because i) statistical power may be lacking to detect significant differences in agronomical traits and ii) there are some unexpected results in the soluble sugars dataset (sustained decrease in glucose and fructose in EPD, and strange behaviour of sucrose at ZT48 for EPD). We need to be sure that it is not due to confusing effects (heterogeneity between the two rotary systems, treatments not harvested on the same day...).

Finally, the graphical layout and statistical analysis of gas exchange could be largely improved. It is almost impossible to see from the figure that "gsw values for CT lettuces increased from ZT0 to ZT2, peaking at a maximum mean value of 0.19 {plus minus} 0.07 moles H2O.m-2.s-1 between ZT3 and ZT4 (figure 2D). Subsequently, gsw values decreased to reach a stable mean value of 0.09 {plus minus} 0.03 moles H2O.m-2.s-1 from ZT11 to ZT16.". A smoothed line (loess or similar) with confidence interval for both treatments should be added. Colour and transparency may also be used for the points. Fig. 5 should be presented right after Fig. 2 (while reviewing the beginning of the results, I thought that the data of Fig. 2 should have been presented as done in Fig. 5, but I did not notice it until I read the discussion where it is first cited). Data of Fig. 3 could also be superimposed with that of Fig. 5 to produce a unique fit for all treatments that could then be compared to individual fits for each treatment.

2. Writing

The text is lengthy at many places, especially considering that the dataset presented here is restricted to one experiment and a few traits. The text should be much more concise. Moreover, the conclusions should not be overstated. Even in the title of the manuscript, the statement "and not by starch depletion" is not clearly established from the data presented here.

Commented [CD7]: Added to the M&M section.

Commented [CD8]: It has been done in a previous experiment, no differences of stomatal conductance were measured between plants that have been under a regular leaf clamping for 8 hours and the control (all cultivation conditions were similar).

Commented [CD9]: Added to the M&M section.

Commented [CD10]: In fact, we have repeated the whole experiment and yields have always been the same, which can be explained by the low amount of light quantity removed. For sugars, data have been corrected

Commented [CD11]: Figures have been modified. They have been simplified and we can now present statistical analyses.

Commented [CD12]: Major modifications have been brought to the Results section in order to shorten it.

Commented [CD13]: We agree with the reviewer and have adapted the manuscript accordingly

Plant Direct
Open Access

Minor comments

- Fig. 1C is misleading because treatments are aligned based on sum of daily light integrals, which progresses differently with real time between treatments, meaning that the interruptions in the axis (double vertical bar) that are used to "resynchronize" treatments graphically should have different lengths between treatments. A real-time representation would help here.

Commented [CD14]: This figure has been removed, as it brings confusion, and the explanations have been simplified.

- line 34: "the circadian rhythm can be defined as a limiting factor for reducing energy consumption in indoor farming." and in the same vein line 556: "Interestingly, the exceptional disruptions of the circadian rhythm allowed to save 5% of lighting time compared to the control, which justifies that circadian rhythm has to be considered to reduce the energy consumption of indoor farming facilities while producing a marketable yield." These sentences are vague, and seem at odds with the conclusion that photosynthesis does not limit growth in these conditions.

Commented [CD15]: Sentences have been removed.

- line 141: "A mutant-free study was carried out to avoid genes shutdown unintended side effects" (abstract and introduction) This sentence is unnecessary: on the one hand the authors don't have to justify themselves to work on a cultivar of a crop species, on the other hand it tends to be irritating for scientists working with mutants.

Commented [CD16]: Removed. It's none of our intent.

- line 158: "Plants were rotating at a speed of 50 minutes per revolution, which provides a centrifugal force of 1,33×10-5 Newton, considered as neglectable." according to which reference? any impact on starch statoliths?

- line 161: "density of 30 plants.m-2" per m2 of ground of per m2 flattened rotary culture?

- line 249: "Two leaves were dried in an oven at 80{degree sign}C for at least 3 days to calculate the dry weight." Unclear. Two leaves of each of the 5 plants? It was not determined on the whole plant like for the FW? It should be clarified.

- line 252: "2D plan" -> "2D plane"

- line 306: "interrelations" -> "correlations"

- line 388: "a decrease of light of approximately 5% was measured in EPD lettuces compared to the control". What is meant with "a decrease of light"? Does this number correspond to the reduction in the sum of DLIs over 31 d (= 1 - 584/610)? Was it measured or computed? It should be reformulated.

- line 457: "Indeed, circadian variations in photosystem II efficiency are difficult to describe because of their dependence on the leaf spot and the species studied (Allen, 2000; Rascher et al., 2001)." Unnecessary, this statement is true for basically every biological process...

- line 462: "In lettuces, we have demonstrated that the photosynthesis is regulated by the circadian rhythm of stomatal conductance." I would not say demonstrated, no experiment has been performed under continuous light, etc...

- line 467: "After an exceptional darkness period of 12 hours, starch residual content of lettuces was equal to 12% of the initial stock, compared to 37% for the control after 8 hours of darkness". Unclear, not in line with the previous sentence. Which point was considered as the initial stock? The argumentation on starch degradation rate is also very unclear: how starch degradation rate can be estimated during the night extension while no data is available at the end of the previous day? I would simply say that starch depletion is observed after a prolongation of the night, while normal starch content is recovered when photoperiodism comes back to its entrainment regime.

Commented [CD17]: Data have been precised, the idea has been reformulated.

- line 502: "these results suggest that starch is the best candidate as to be indicator of the circadian rhythm amongst the studied sugars." sugars -> carbohydrates; this sentence is overstated, not only there is no apparent link between starch and the clock in the data presented here, but also sucrose looks probably more interesting (but its dynamics has to be independently replicated).

Reviewer #2:

Comments on 'Photosynthesis is led by stomatal circadian rhythm and not by starch depletion after a long 2 period of darkness'.
General Comments
The manuscript focuses on Lettuce response to an extended night period. Authors looked into photosynthetic changes during the light period right after an extended night and compared them to a control treatment of constant day/night periods. They complement this response with several morphological variables measured at the end of the growth cycle. They found slight changes in net CO2 assimilation, no changes in stomatal conductance, and no differences between morphological variables. In this context they argue that the circadian rhythm controls stomatal conductance and the latter controls net assimilation. The literature on this topic is mixed, and many suggest that changes are driven by carbohydrate synthesis. The authors also suggest that this extended night period could increase the energy efficiency of the system, without affecting yields. I am positive the topic would be of interest for the readers of Plant Direct, however, the manuscript is not yet ready for publication and major changes need to be addressed.
Overall, the manuscript reads with ease, but it presents majors flaws that need to be addressed.
The introduction is merely descriptive and does a poor job guiding the reader to a knowledge gap. Authors spend most of the introduction describing already-known processes and barely comment on the scientific question they are trying to answer. In addition, multiple sentences read disconnected and lack proper reference support. Also, the introduction does not state any clear objectives or hypothesis, which makes reviewing the manuscript extremely hard.

Commented [CD18]: We agree with the reviewer and have adapted the manuscript accordingly.

The methods section does not provide enough detail on the experimental design, the growth system,

and the methodology and analysis of some variables. A better description of the rotary culture system is much needed, what is the purpose of the rotation? is it rotating vertically or horizontally? Where is the light source? What is the CO2 concentration? was it controlled?

And, most importantly, how many were used in the experiment? This brings a very important point, the number of replicates is not very clear, based on comments in the results section, it seems it only one per treatment, is this right? If so, it could be a major flaw as it would not capture the variability. Authors must discuss this topic and how they overcome this limitation.

More detail is also need in how the Licor measurements were collected and analyzed. The authors said that photosynthesis was measured continuously for 16hr, and that measurements were taken manually on 10 plants. Saying 'continuously' could mean that they did not unclamp the leaf and were measuring using the Dynamic Assimilation Technique. If that is the case, and if they measured 10 plants, does it mean you used 20 licors? How many instruments did you use?

Finally, what was the statistical analysis used for photosynthesis data?

The Results section is also very descriptive and tends to repeat the data. I would encourage the authors to be more clear when presenting percentage changes, and to add p-values when comparing treatments. Also, Figures 2, 3 and 5 have serious issues. First, do not repeat data, there is no reason to present data of each treatment separately (A, B) and then combined (C), just present C. Also figure E should be split. Each variable should be its own plot.

Also is it possible to add some type of trend for each treatment, it would make it much easier to see the patterns. Also, what does each point represent? You mentioned there are 10 plants and two leaves per plant, is data averaged in some way? Could they be averaged over reps? As presented, the data seem noisy which makes it hard to see the trends between treatments.

In my opinion the discussion does not provide enough support to the main idea of the manuscript that the circadian rhythm control stomatal conductance and the later controls photosynthesis. References are scarce, a bit outdated, and mainly on Arabidopsis. I understand the value of a model species, but work done in lettuce or other leafy greens would be more informative.

Another important point is that a big piece of evidence for support the circadian control on stomatal conductance and assimilation is a ~ 6% decrease in assimilation. I think this has three limitations; first, 6% is not a large difference mainly considering the large variability in your dataset, second, the average may not be the most indicative parameter, instead total daily assimilation could provide a better description of the patterns, and third, you also report and increase in A_net in the early hours of the light period, but it is not incorporated in the discussion.

Another aspect that is missing in the discussion is whether the extended night period is enough to cause a change in the circadian rhythm. It could be that plants coming out of the 12hr night period did not acclimate to the change and they responded as the control.

These limitations make it very hard to conceptually review the manuscript and must be addressed. I am also attaching a pdf with in-line comments in the manuscript and figures. Some may be more relevant than others, but please have a look at them.

Commented [CD19]: Information added.

Commented [CD20]: The same rotative cultivation system was used to do the experimentation one after another in order to suppress all variability induced by differences between cultivation systems. Temporal differences between experiments are not problematic as we are in a totally controlled environment.

Commented [CD21]: This part of the M&M has been changed, we used only 1 LiCor and we studied 20 independent leaves one after another. Each measure took approximately 3 to 5 minutes so that each plant were approximately studied every hour.

Commented [CD22]: We have modified the figures in order to be able to present statistical analyses. Photosynthesis analyses has been totally changed.

Commented [CD23]: Results section has been drastically shortened and figures have been modified.

Commented [CD24]: Yes, we have averaged all the points by periods after the beginning of the lighting period.

Commented [CD25]: Discussion has been changed, according to the overall idea of the article. We have made the mistake to focus on the theorical knowledge brought, which is really scarce compared to the applied conclusion that we aimed to present.

Commented [CD26]: Added to discussion, or considering into the Result section.

Commented [CD27]: Added to discussion.

Plant Direct
Open Access

Response to Reviewer Round 2 :

Response to reviewers

All author responses to reviewers are formatted in italicized text and presented in green.

-- Reviewer comments:
Reviewer #1:

I have reviewed again the manuscript by Dresch et al.

The major issue of reproducibility remains. There is only one independent experiment for each treatment (control and EPD), and each treatment was assessed in a separate experiment. Can we detect a yield reduction with more statistical power? Are Glc and Fru contents always lower in the EPD treatment (potentially underlying the preservation of yield in spite of the decrease in photosynthesis)? These questions will remain if only one experiment is presented. It is claimed in the rebuttal that "In fact, we have repeated the whole experiment, and yields have always been the same" but data are not shown or even mentioned in the manuscript. Alternatively, other light scenarios may have been explored to challenge the proposed model - but the findings are based on a single experiment.

The experiment was replicated under identical conditions, as it was conducted in a controlled environment agricultural setting. The results obtained enhanced the robustness and reliability of the data.

The text has been clarified and made more concise as recommended. While I appreciate these efforts, there is still a large imbalance between the results (about 700 words) and the discussion (about 1800 words). Some parts are still hard to follow (e.g. lines 54-67, 369-384). English needs revision as well.

Discussion has been synthesized and the English has been revised.

The figures have been improved, yet I regret that gas exchange measurements were binned into 4 discrete periods because it blurs the actual temporal patterns - making the phase shift much less perceptible. A similar layout like Fig. 5D and E would have been more appropriate (with statistics on binned data possibly added in Supp Data).

Plant Direct
Open Access

All figures have been revised with the addition of the new experiment.

As mentioned in your rebuttal, the Suc data have been corrected. Comparing the data between the previous and actual versions, it seems that two points have been switched (40 and 48h for EPD lettuces). Such a switch would have deserved an explanation. It is a pity that the mistake was not detected before, given the important implications for the interpretation.

Sucrose data of the new experiment have confirmed previous corrections.

Lines 327-352: the discussion about potential adjustment of starch degradation rate does not make sense. Gibon et al. (2004) showed that starch degradation rate slows down when the night is extended due to an anticipated start of the night. In your manuscript, night is extended due to a delay in the start of the next day, so there is no way for the plant to anticipate this change - resulting in a mismatch between stomatal opening and light unavailability. Moreover, the starch degradation rates mentioned here are computed based on data not shown.

Discussion has been revised.

Reviewer #2:

I appreciate the authors for taking care of my general comments. The manuscript has improved in this round of revisions.
That said, I think the manuscript still has two main pitfalls. First, the introduction does not do a good job introducing the knowledge gap. Authors spend a lot of space describing processes that are already well known and understood but fail to introduce how these processes interact among each other in the context of their research. To be clear, more details need to be provided about the interaction between photoperiod, circadian rhythm, photosynthesis (CO2 assimilation and stomatal conductance) and carbohydrates.

Introduction has been revised.

For the second point, I would encourage the authors to think of the results in terms of the source-sink balance of the leaf. See below my comments on how the initial increase in assimilation could be driven by the depleted reserves of the extended night, and it may only take a few hours for the plant to restore the carbohydrate balance. As is, it is very biased toward the changes in stomatal conductance, but there is not much support from previous studies or from their own results.

The idea has been added to the discussion.

Also see below the detailed comments. Many of them were already in the pdf of the previous version. I hope having them here helps the authors to include them.
L 1: Phase shift is ambiguous

The title has been revised.

L 47: The intro is missing key points of the research and add too much detail in already established knowledge. I suggest adding more detail into how photoperiod affects photosynthesis, whether these effects are driven by stomatal conductance, circadian cycle and/or source sink balance of carbohydrates. After reading your discussion, these the three main topics you discussed and are not introduced.

The introduction has been revised to better fit the discussion.

Also, it's known that plants adapt to constat changes in the photoperiod, but what is it known about plants adapting to more complex cycles, such as the one in your study? This is very important and should be included in the intro.

This is a key part of the objective of the study, which has been clarified.

L49 - 85% of what?
L50 - Don't need this, you aren't going to talk about it later.
L52 - I like how you reshaped this paragraph. I think it would be important to highlight the knowledge gap you are trying to fill. A sentence like this makes it seem that there is no knowledge gap and there is no need to do you study.
L55 Add hyphen.
L55 - instead of multiplied by two use "doubled"
L63 - Not sure what you are trying to say.
L 69 - change for 'and'

Introduction has been revised.

L 70 - I understand the relevance of photosynthesis to this research but there is absolutely no point in describing such a well-known process here. I mentioned this in the previous round and the problem persists. Instead of spending half a page in photosynthesis, you should introduce how photosynthesis could be affected by circadian and photoperiod changes.

Photosynthesis detailed presentation has been removed.

L 86 - Same as with the previous paragraph, this is merely descriptive and is not related to the research

Plant Direct Open Access

question.
L105 - Two points here:
1- Does no one done any research on extending the night period? This the research you should be mentioning in the intro.

No similar study has been found in the existing literature. Other studies that extend the night period do so either daily or through irregular light reduction patterns with a primary focus on optimizing electricity consumption.

2 - This needs further clarification, as it reads it's a bit confusing how is extending the night period an energy saving opportunity. If you are keeping the lights on for the same time then the energy consumption would be equal. The key point (I think) is that plants grew for the same number of days.

Scientific question has been clarified.

L 113 Somewhere you must explain how treatments were implemented. Based on the response to the reviewers, you mentioned that you did one treatment and then the other. You must explain this and discuss that it doesn't affect the measure. You run multiple runs of this, you should also present the data
L152 It would be very useful if you could indicate in this table when the different samples took place. Also, based on the figure is not clear to me where the energy savings are coming from. Despite EPD plants having a total of two longer nights, it seems these extra 8 hours are not removed from the total length of the period. Then it seems that the EPD would end their growing period later. I made a point in the objective about this. This should be explained in further detail and represented accordingly.
L 160 how could be consistently equal if they are not controlled?

Information on energy savings has been excluded to maintain focus on the key message regarding plant physiology.

L164 All figures should also include the days after transplant.
L168 just to be clear, you have an n = 1. all plants are subsamples of the single treatment. This must be discussed in the discussion or methods. Change n in your figures and say the number of plants.

We added information about the plants and leaves studied in each figure legend related to photosynthesis and stomatal conductance. 'n' is specified to precise the number of observations for each treatment in each experiment.

L 225 any time you mentioned significant, you must say the pvalue at which you consider it. Also, provide the p-values of the comparisons when you mentioned them on the text.

We added a supplemental table in which every p-values of each test are precised.

L226 Also, mention the functions and packages you used.
L 226 I don't think this is the right way to refer to R studio. citation() in Rstudio gives:
R Core Team (2022). R: A language and environment for statistical computing. R Foundation for Statistical. Computing, Vienna, Austria. URL https://www.R-project.org/.
L 232 Addressing morphology from a picture is challenging. As the other reviewer mentioned, leaf area would be a better index.
L236 here and for the rest of the manuscript; avoid single-sentence paragraph.
L 239 It seems a bit odd to represent the time with such a large interval. Did it take you 4 hours to measure all plants? if so, you may need to do some corrections by leaf. If not, present the average of the times

Figures have been modified.

L 242 After reading the discussion, this sentence seems to be very important. You must be more clear about how this 6.2% was calculated. Was it the average during the day? I think there is also a lot of value in analyzing this data by each time point. Based on the graphs it seems that it only takes the leaf the first hours of the day to restore the imbalance.

Precision have been added in the discussion section.

L 260 You only have data for the beginning and end of the light phases, then you can't say what happened during the rest of the photoperiod. Saying that is stables is an overstatement, and can't be supported with your measurements. It could be that they go up in concentration and then return to the initial values
L 263 what's the pvalue of this test, I see that they tend to increase in the graph but the deviation is pretty large. To be honest, the lack of p-values is starting to make me doubt about the validity of your tests. Please add pvalues and more detail in the stat section

We understand that. However, for photosynthetic parameters, we have a high robustness of statistical tests used. Even low differences are thus detected as significant by the test.

We are concerned that including all p-values in the figures would reduce their readability. Therefore, we have provided a supplementary table detailing the p-values for each test.
L 288 Daily usually refers to the total amount of net assimilation accumulated during the day. Please use a different term.

We defined more explicitly the calculation method in the material and methods section. We indeed calculated the total amount of net assimilation accumulated during the day.

L289 Need references to support this.

L 290 The link between anet and gsw seems to be a key finding of you research but it is poorly described. please expand on the reasoning and how your data supports this. I personally don't think you have the data to support that is gsw that drives the decline in Anet, and would like to read more about your explanation.

This point has been clarified using a mathematical approach, supported by a supplementary figure.

L 294 You don't need to present the pvalue of another research. The R2 could be more informative. That said, you MUST report the pvalue of your tests.
L 297 why do you define stomatal conductance now? do it the first time you mentioned it.
L298 This is a very important point of your manuscript. I would suggest supporting it with other studies. Also, I would include part of the main points in the introduction.
One aspect that is missing with this explanation is the rates of Anet being higher during the first measurement. Your data show a clear correspondence between increased gsw and Anet but your hypothesis doesn't explain the increase in Anet.

Introduction has been revised and figures have been modified with a new experiment. Thus, the presentation of the information has been revised.

If we look at this trend from source-sink perspective, increased A net could be driven by the lower sucrose levels. Unfortunately, you don't have a diurnal course of carbohydrates. Based only on Anet, and assuming a direct correspondence with sucrose. It could be that it only takes ~4hours for the EPD plants to potentially replete the reserves to CT levels, and from there on, the source-sink balance is restored and photosynthesis goes at the same rate.
L 303 See my previous comment, I think these minor differences could be what EPD plants need to restore the source sink balance.
L 328 does 'which' refer to photosynthesis or the circadian rhythm
L 329 had
L 336 why not? the increased Anet at the beginning of the day could be what drove the increased starch accumulation.

A supplementary figure has been added to provide detailed information on starch accumulation. Additionally, measurements of starch accumulation throughout the day have been included in supplemental figure.

L 339 Could
L 341 How could this translate to your study? in your case the plants experience the increased light period 1 every 3 days, how would the plant know that it's experiencing an extended darkness period?
L 347 but you just said that it was adjusted in arabidopsis?
L 357 what this means?

L 359 your study? or the one referenced? please be clear
L 362 in which species?
L 373 why are you making the decrease in sugars being NOT correlated with Photosynthesis? From a source sink perspective, it should be the opposite. A
L 375 but it was higher in the first time of the day??? It may be all the plant needs to readjust
L 376 This needs to be further discussed. There is lot of literature showing the opposite, in fact trio-phosphate utilization is one the main limitations of photosynthesis
L 381 Which other tissues? And how this relates to your study

This part of the discussion has been reshaped to add clarity.

L 385 Your data doesn't support this statement. You saw that the without a change in starch there was not change in yield, but you didn't do any experiment to test show this causation.
L 387 This paragraph is a bit confusing, it starts mentioning how assimilation doesn't change and then talks about fresh weight. I think a big missing component in the intro is how changes in photoperiod interact with photosynthesis and ultimately biomass. Something about the effects of sucrose and starch and the imbalance of the source-sink system may affect biomass.
L 388 cite one of your figures/tables,
L 393 is there a missing word here??
L 404 This reasoning is missing the fact that both the 6% decline in Anet and the decreased in carbohydrates are diurnal averages after the change photoperiod. It could well be that the EPD plants readjust their source sink balance within the first hours of the day and that is enough to compensate for the declines in carbs.
Another aspect that is missing in here and in the discussion is the idea of 'storage carbohydrate' the fact that glucose and fructose are constant at the beginning and end of the day may say that this are produced and consumed during the day. Sucrose and starch change increase during the day suggesting this are the storage carbohydrates. These are more relevant to the source-sink balance of the leaf. In both cases they are further depleted right after the extended night but replenished to control levels within one day. it seems that within the first few hours of the day (the first timepoint) leaves are able to replenish the reserves and then they returned to control dynamics.

This idea has been added to the discussion.

Specific discussion on yield has been combined with the photosynthesis in order to shorten and clarify the discussion part.

L 413 This just repeats the previous sentence. It would be good to add more support,
L 415 Daily photosynthesis is commonly used to refer to the integration of net assimilation over the day. I suggested this on my previous review. Please use a different term.
L 423 Where does this 5% comes from? It should be explained somewhere or if the math is simple added

Plant Direct
Open Access

between brackets here
L 474 This table is very interesting, and it's barely mentioned in the results. Do you have any hypothesis of why EPD plants are almost 15% more efficient than control?

EPD lettuces had the same yield as the control ones, but in the meantime, after the same DAT, they had 5% less light (due to the periodic 4-hour extent of the dark period). Moreover, irrigation was solely done during the lighting period, and thus 5% less water was given to EPD lettuces.

Thus, we used fewer resources and had the same final yield, which increases efficiencies values. However, this table has been removed to maintain focus on the most important findings of the experiment.

Good Job!! The manuscript has improved from the previous version. I think adding these will notably increase the quality.

Response to Reviewer Round 3 :

Response to reviewers

All author responses to reviewers are formatted in italicized text and presented in green.

-- Editor comments:

Thank you for adding the data for an additional experiment and incorporating the suggestions of the two reviewers.

The main issue that needs to be addressed are the apparent discrepancies on how these experiments were performed as shown in the depictions of dark and light periods in Figures 1B and 4. Here are a couple of more comments and suggestions that should help further improve the manuscript.

Line 196: The phrase 'repeated period of darkness of 12 hours', is slightly confusing, since the key change is the elongation of the night by 4 h which causes a phase delay. More informative would be "... by repeated elongations of the night period'. There seem to be some discrepancies between Figure 1B and Figure 4 on how the treatments were done, please see my comment below.

Title of this result part has been corrected.

Line 200: should be ' differences in fresh weight and dry weight between the two conditions were only detected...'. Since these differences were not reproducible between experiments, it would be more informative to the reader to write that not reproducible differences in biomass were detected between the two treatments.

Sentences have been corrected, and ideas have been added.

It would be useful to indicate in Figure 1B what the authors define as 'Lighting period 1' and 'Lighting period 2' and 'Lighting period 3'.

Figure 1B has been modified, lighting periods have been specified.

The 3rd, or 4th panel in Figures 2A/B and 3A/B, are not very informative. They show the 'CT' treatments as if they were measured in the dark (although that is clarified in the legend). It is rather confusing. Based on Figure 1 than the EPD plants would have been subjected to a phase shift, but that is not how Figure 4 shading is depicted.

Figures 2 and 3 have been updated, with the black background removed to reduce any confusion. Lighting periods have also been added to Figure 4. In the legend, we've clarified that a double slash in the EPD modality indicates the omission of 4 hours of lighting, for better understanding. While the phase shift is real, this representation has been chosen to more clearly highlight the statistical test with an asterisk and to prevent any confusion.

Here again, for the data shown in Figures 2 and 3, Experiments 1 and 2 do not show consistent differences between CT and EPD treatments. Therefore it is only distracting to the reader describe in detail differences that are not consistent between experiments.

The descriptions of figures 2 and 3 have been combined and less details have been provided on non-consistent differences.

It is unclear what which data is shown in each panel. The EPD LN treatments have only one time point. It might be clearer to indicate the lighting period as for figures 2 and 3. There are again only very few, if any, differences between the experiments.

Lighting periods have been added and are now consistent with those presented in figure 1B, to ensure clarity.

Line 263: The authors write that the EPD treatment "affected the daily trend of net photosynthesis". I do not think there are any consistent trends between Experiment 1 and Experiment 2 (Figures 2 and 3). I also do not think the data shown provides any evidence for or against circadian control.

The sentence has been modified; affected has been replaced by “induced a 4-hour shift”, which is more directly justified by the conducted measurements.

Line 281: The authors write that 'due to the extension of the night, only 4 hours of maximal net photosynthesis remained'. I am not sure what is meant by this. According to Figure 1B, the extension of the night leads to a phase shift, the next light period, after the extended dark, is also 16 h. However, I see that that is not the case in the Figure 4, in which the light period after the extended dark is only of 12 h. This needs to be clarified. I cannot see any consistent decrease in carbon assimilation in EPN conditions across both experiments in any of the lighting periods. As the reviewers indicated about previous versions of the manuscript is unclear how the decrease in the daily photosynthesis of EPD lettuces was calculated.

Figure 1B and figure 4 have been modified to avoid confusion. Moreover, in figure 4, we've clarified that a double slash in the EPD modality indicates the omission of 4 hours of lighting, for better understanding.

To avoid any further confusion, we specified the calculation method and provided the amount of daily net photosynthesis in $\mu mol.m^{-2}.d^{-1}$*. Moreover, all net photosynthesis and stomatal conductance, as well as the daily photosynthesis calculation have been added to the supplementary data (first data sheet).*

Line 297. A main difference of the described experiments and other studies is that in this one the plants are treated to a 4 hour 'jet lag' every 3 days. Other experiments grew plants on different T-cycles constantly. So it seems that a more 'sporadic' phase change does not affect the plants strongly.
This has been added to the discussion.

Other comments:
Numbers: decimals are indicate with "." not ","
units: please don't use "." such as umol.m (=micromol per meter), just use a space or "x", but please be consistent, don't use space and 'x'.

All units have been corrected. Figures have also been corrected.

Line 151: should be 'light' not 'lighting'
Line 156: explain HPAEC abbreviation

Corrections have been done.

Line 178: please provide full protocol or reference. This protocol does not allow reproducibility.

Details of the protocol used have been added.

It is unclear how fresh and dry weight were measured. Were these assays distructive? i.e. were whole plants harvested?. In Line 189 it said that only 2 leaves were dried, is that sufficient to estimate the dry weight of the whole plant?

Yes fresh weight measurement are destructive, whole plants were harvested. Details have been added in the M&M section. In fact, 2 leaves were dried, but in leafy vegetables, this is possible because all the organs of interest (leaves) have a very similar water content. We verified this assertion before the experiment. In addition, we used a representative portion of the plant.
